# Effects of Prednisolone on Disease Progression in Antiretroviral-Untreated HIV Infection: A 2-Year Randomized, Double-Blind Placebo-Controlled Clinical Trial

**DOI:** 10.1371/journal.pone.0146678

**Published:** 2016-01-26

**Authors:** Christa Kasang, Samuel Kalluvya, Charles Majinge, Gilbert Kongola, Mathias Mlewa, Irene Massawe, Rogatus Kabyemera, Kinanga Magambo, Albrecht Ulmer, Hartwig Klinker, Eva Gschmack, Anne Horn, Eleni Koutsilieri, Wolfgang Preiser, Daniela Hofmann, Johannes Hain, Andreas Müller, Lars Dölken, Benedikt Weissbrich, Axel Rethwilm, August Stich, Carsten Scheller

**Affiliations:** 1 University of Würzburg, Institute of Virology and Immunobiology, Versbacher Strasse 7, 97078 Würzburg, Germany; 2 Bugando Medical Centre, P.O. Box 1370, Mwanza, Tanzania; 3 Catholic University of Health and Allied Sciences, P.O. Box 1464, Mwanza, Tanzania; 4 Medical Mission Institute, Salvatorstrasse 7, 97067 Würzburg, Germany; 5 University Clinic of Würzburg, Division of Infectious Diseases, Dept. of Internal Medicine, Josef-Schneider-Str. 2, 97080 Würzburg, Germany; 6 HIV-Intensive Care Unit, Schwabstr. 26, 70197 Stuttgart, Germany; 7 Division of Medical Virology, National Health Laboratory Service / University of Stellenbosch, Tygerberg 7505, South Africa; 8 University of Würzburg, Institute of Mathematics and Informatics, Chair of Mathematics VIII (Statistics), Am Hubland, 97074 Würzburg, Germany; Rush University, UNITED STATES

## Abstract

**Background:**

HIV-disease progression correlates with immune activation. Here we investigated whether corticosteroid treatment can attenuate HIV disease progression in antiretroviral-untreated patients.

**Methods:**

Double-blind, placebo-controlled randomized clinical trial including 326 HIV-patients in a resource-limited setting in Tanzania (clinicaltrials.gov NCT01299948). Inclusion criteria were a CD4 count above 300 cells/μl, the absence of AIDS-defining symptoms and an ART-naïve therapy status. Study participants received 5 mg prednisolone per day or placebo for 2 years. Primary endpoint was time to progression to an AIDS-defining condition or to a CD4-count below 200 cells/μl.

**Results:**

No significant change in progression towards the primary endpoint was observed in the intent-to-treat (ITT) analysis (19 cases with prednisolone versus 28 cases with placebo, p = 0.1407). In a per-protocol (PP)-analysis, 13 versus 24 study participants progressed to the primary study endpoint (p = 0.0741). Secondary endpoints: Prednisolone-treatment decreased immune activation (sCD14, suPAR, CD38/HLA-DR/CD8+) and increased CD4-counts (+77.42 ± 5.70 cells/μl compared to -37.42 ± 10.77 cells/μl under placebo, p < 0.0001). Treatment with prednisolone was associated with a 3.2-fold increase in HIV viral load (p < 0.0001). In a post-hoc analysis stratifying for sex, females treated with prednisolone progressed significantly slower to the primary study endpoint than females treated with placebo (ITT-analysis: 11 versus 21 cases, p = 0.0567; PP-analysis: 5 versus 18 cases, p = 0.0051): No changes in disease progression were observed in men.

**Conclusions:**

This study could not detect any significant effects of prednisolone on disease progression in antiretroviral-untreated HIV infection within the intent-to-treat population. However, significant effects were observed on CD4 counts, immune activation and HIV viral load. This study contributes to a better understanding of the role of immune activation in the pathogenesis of HIV infection.

**Trial Registration:**

ClinicalTrials.gov NCT01299948

## Introduction

Untreated HIV infection causes a progressive loss of helper T-cells that results, after an average period of about ten years of ongoing virus replication, in immunodeficiency and ultimately AIDS. HIV infection is also characterized by a general, chronic immune activation that persists throughout the entire course of infection and that is tightly linked to disease progression. Patients with higher levels of general immune activation progress more rapidly to T-cell loss and AIDS than individuals with lower levels [1–5]. For example, long-term non-progressors, who exhibit reduced levels of immune activation, do not progress to AIDS for a long time, despite ongoing virus replication [6]. In contrast, elite controllers, who are able to control virus replication below detection limits, may still progress to AIDS if they have elevated levels of T-cell activation [7]. Even in the presence of highly-active antiretroviral therapy (HAART), increased immune activation correlates with higher morbidity and mortality [5, 8–10], a phenomenon that peaks in a special group of patients, the “immunological nonresponders”, who fail to regain normal T-cell counts despite complete suppression of virus replication under HAART [11]. The same correlation has been observed in SIV-infected monkeys: The natural hosts of SIV, African monkeys, remain relatively unaffected by SIV infection and exhibit neither chronic immune activation nor progression to AIDS despite high-level virus replication, whereas Asian monkeys develop chronic immune activation and AIDS (reviewed in [12]).

A number of clinical studies describe the importance and predictive value of immune activation for mortality and non-AIDS comorbidities in HIV infection, including, but not restricted to, the SMART (acronym for “strategies for management of antiretroviral therapy”), SCOPE (acronym for “observational study of the consequences of the protease inhibitor era”) and LSOCA (acronym for “longitudinal study of ocular complications of AIDS”) cohort studies [5, 13–15]. Consequently, there is a profound interest in immunomodulatory adjunct therapies that target this immune activation. Different therapeutic strategies have been tested, including probiotics [16], COX-2 inhibitors [17, 18] and statins [19, 20] (a more extensive review on this subject is presented in [21]), and all have shown efficacy in terms of reducing HIV-immune activation. Corticosteroids belong to another promising substance class to reduce HIV immune activation. In a prospective but uncontrolled clinical trial, treatment with prednisolone (0.3–0.5 mg/kg body weight) increased CD4 counts by 119 cells/μl within one year without concomitant changes in HIV viral load [22, 23].

To answer the question whether the reduction of immune activation translates into clinical benefit we need studies with clinical endpoints. Here we report on an investigator-initiated, interventional, two-year, randomized, placebo-controlled prospective clinical trial in Tanzania in which we aimed to reduce HIV immune activation with prednisolone in order to monitor the effects of this intervention on HIV disease progression. When we started the trial, the local access to HAART was still very limited, so that the Tanzanian National Treatment Guidelines recommended that asymptomatic HIV patients remained without antiretroviral treatment when the CD4 count is above 200, a situation that changed only recently in 2012. This relatively late onset of HAART during the study period not only defined the need for cheap and readily available treatment alternatives, but also allowed us to reach a clinical endpoint (i.e. the onset of AIDS-defining opportunistic infections) in order to address the question of whether a reduction of HIV immune activation translates into clinical benefit.

## Materials and Methods

### Ethics statement

The study was performed according to the declaration of Helsinki and approved by the ethics committees of the Tanzanian National Institute for Medical Research (NIMR) and the Catholic University of Health & Allied Sciences, Mwanza. Patient information was provided in Kisuaheli and in English. Written informed consent was obtained from all study participants. The study protocol will be made available as Supporting Information (S1 File). The study was supervised by a data safety monitoring board (DSMB) whose members gave a written report to the Tanzanian TFDA (Tanzania Food and Drugs Authority) on an annual basis.

### Trial registration

The trial was registered at clinicaltrials.gov (NCT01299948). The trial was registered on February 18^th^, 2011 i.e. after enrollment has begun (FPFV 06/2007). At the time when the trial was started, the initiators of this study were unfortunately unaware of the policy of the International Committee of Medical Journal Editors (ICMJE), which requires prospective registration of all interventional clinical trials. As soon as we became aware of this policy, we registered the trial. The authors confirm that all ongoing and related trials for this drug/intervention are registered.

### Trial design

An investigator-initiated, two-year, randomized, double-blind, placebo-controlled, single-center trial studying the effects of prednisolone *per os* in a daily dose of 5 mg on HIV disease progression during the early phase HIV infection (CDC stage A1, A2, B2, B2). A total of 326 patients were randomized; 163 study participants received placebo and 163 study participants received prednisolone. Clinical and laboratory evaluations were performed before start of treatment (baseline) and at months 1, 2, 3, 4, 5, 6, 9, 12, 15, 18, 21 and 24. FPFV (first patient first visit) was in 06/2007, and LPLV (last patient last visit) was in 03/2011. The consort checklist (S1 Checklist), the study protocol (S1 File) and and the minimal data set (S1 Dataset) are available as Supporting Information to this publication.

### Participants

Patients eligible for the study fulfilled the following criteria: 18 years of age or older, proven HIV infection according to WHO guidelines, CD4 counts above 300 cells/μl, negative pregnancy test, and a CDC stage of A1, A2, B1 or B2. Patients with prior antiretroviral medication, active tuberculosis, abnormal laboratory results (glucose level >160 mg/dl, liver enzymes AST and/or ALT ≥ 1,5 x ULN, bilirubin ≥ 4 x ULN, alkaline phosphatase ≥ 5 x ULN, creatinine ≥ 2 mg/dl) or other serious diseases, including psychiatric disorders, were excluded from the study. The study was performed at the Bugando Medical Center (BMC) in Mwanza, Tanzania.

### Interventions

Study participants received prednisolone *per os* in a daily dose of 5 mg or placebo self-administered by the study participants at home. At each study visit, study participants received sufficient study drug for the period until the next visit. Study participants were asked to return unused study drug at the next study visit to assess study drug compliance. The protocol required a minimum drug adherence of greater than 80%. Patients falling below this minimal drug adherence were not excluded from the study but received intensified counseling on the proper dosage of the study drug.

### Outcomes

The primary endpoint was time to progression to one of the following events: a) death, b) progression to CDC stage A3 or B3 (i.e. CD4 T-cell count < 200) or progression to CDC stage C (AIDS-defining illnesses, including tuberculosis). Following progression, study drug medication was discontinued and study participants received standard antiretroviral medication. At the beginning of the study (2007) this endpoint was consistent with the “National Guidelines for the Clinical Management of HIV/AIDS–Tanzania, 2^nd^ edition 2005” of the National AIDS Control Programme, which recommended HAART at CD4 counts < 200 or for WHO stage 4 disease. In 2009 the national Tanzanian guidelines were updated (3^rd^ edtion, Feb. 2009) and initiation of HAART was recommended at a) CD4 counts < 200, or at b) CD4 < 350 in combination with a WHO stage-3 disease, or at c) WHO stage 4, regardless of CD4 count. Due to limitations in supply of antiretroviral medication this recommendation was implemented at the study site only since 2011 and the endpoint criteria in the study protocol were therefore left unchanged. In a total of 4 cases in this study these updated criteria were applied to put study participants on antiretroviral medication. In 2012 the most recent update was released, which recommends treatment at WHO stages 3 and 4, regardless of CD4 count, or at a CD4 count < 350. By this time, the study had already been completed (LPLV 03/2011).

### Sample size

Sample size was calculated according to published data on the effects of prednisolone on CD4 counts [22–25]. This data indicated that the probability of progression to the primary study endpoint in a two-years period among controls is 0.791. If the true probability of progression among cases on treatment is 0.625, we would have needed to study 199 participants in the prednisolone arm versus 199 participants in the placebo arm (as determined by NQuery 3.0 Test, calculated with PS Power and Sample Size Calculations by William D. Dupont and Walton D. Plummer) to be able to reject the null hypothesis that the progression rates for cases on treatment and controls are equal with a probability (power) of 0.8. The type I error probability associated with this test of this null hypothesis is 0.05. The study was therefore designed to recruit 200 versus 200 participants. Towards the end of the 12-months recruitment period it became clear that this number could not be met. The investigators decided to recalculate the needed sample size using a less-robust test (an uncorrected chi-squared statistic) to reevaluate this null hypothesis. Using this test, 117 participants in the placebo arm versus 117 participants in the prednisolone arm were sufficient to reject the null hypothesis. It was therefore decided to stop recruitment at the scheduled time point at then 163 versus 163 study participants.

### Randomization and blinding

We used a computer-generated randomization sequence with a block size of eight to generate a list that assigned consecutive patient numbers in a 1:1 ratio to active drug or placebo. The randomization list, which was deposited at one of the members of the independent scientific board of the study (Prof. H. Klinker, Germany), was not accessible to the investigators; also the block-size remained unknown to the investigators. The study medication was labeled by the manufacturing company (details see below) with the study numbers assigned to prednisolone or placebo. Patients who entered the study were assigned to their respective study numbers (and respective study treatment) in chronological order. The randomization process was supervised by “Deutsches Medikamentenhilfswerk action medeor e.V.”. Treatment was double-blinded during the whole study period, i.e. neither the treating physicians nor the patients did know what kind of study medication (placebo or active drug) was being allocated.

### Study medication

Study medication was prepared by Regal Pharmaceutical Ltd (Nairobi, Kenya). Each tablet contained 5 mg prednisolone (active component) or lactose (placebo) with identical shape and color (white). Regal Pharmaceutical Ltd also labeled the study medication with the respective study patient numbers from the randomization list according to its content. Production was supervised by “Deutsches Medikamentenhilfswerk action medeor e.V.”.

### Clinical evaluation

Study participants were clinically examined at each study visit for vital signs, HIV staging according to CDC criteria, and pregnancy (urine test). Tuberculosis was tested by sputum test every three months and by chest X-ray every six months. Malaria was diagnosed on clinical grounds and generally confirmed by microscopic analysis of blood films. Study participants diagnosed with malaria received artemether/lumefantrine and, in severe cases, quinine.

### Statistical analysis

Study data were subjected to *intention-to-treat* (ITT) analysis (163 versus 163 study participants). Estimated proportions of study participants surviving without progression were calculated using the Kaplan-Meier method. Differences between two survival curves were tested by log-rank (Mantel-Cox) tests. ANOVA with repeated measures was used to assess differences between the two treatment arms with respect to CD4 counts. Average changes in CD4 count per year were calculated using linear regression of changes in CD4 count relative to baseline in both treatment groups over the two-year study period. We also performed a *per protocol* (PP) analysis, in which we included only study participants who had an average minimum study drug compliance of greater than 80%, i.e. the average study drug adherence of all pill counts combined had to be greater than 80%. Pill counts of unused study drugs were performed at 3-months intervals at the study visits. The PP population in this manuscript also includes the study participants who were lost to follow-up and these subjects were censored in the Kaplan-Meier analysis. Technically, the PP analysis performed in this manuscript is therefore a “modified ITT analysis”, as PP analyses usually exclude the data from participants who missed one or more study visits. Statistics and Graphics were performed using GraphPad Prism 6.0 for Mac OSX.

### Laboratory analyses

CD4 counts were determined at Bugando Medical Center, Tanzania, using a FACScan or a FACS Calibur cytometer (Becton & Dickinson) during clinical routine. All other laboratory analyses were performed after storage (-20°C) and dry ice-cooled shipment of the samples at the University of Würzburg, Germany. HIV viral load (VL) was determined from plasma for all study participants who continued study drug medication until month 12 at baseline and at month 12 using a COBAS^**®**^ AmpliPrep Instument (Roche Diagnostics). ELISAs to detect plasma levels of sCD14 (Diaclone), and suPAR (ViroGates) were performed according to the manufacturer’s instructions. For flow cytometric analysis of frozen PBMC, cells were stained with with antibodies directed at CD3 (labeled with FITC) and CD8+ (labeled with PerCP) and counterstained with anti-CD38-PE and anti-HLA-DR (APC) (all antibodies from were purchased from BD Biosciences, Heidelberg, Germany) according to the “lysis no wash” protocol (BD Biosciences). Cells were analyzed by flow cytometry using a FACS-Calibur four-colour flow cytometer (Becton Dickinson). We analyzed all events that fell into the FSC/SSC area in which living lymphocytes usually reside; we did not use any viability dye to discriminate between live or dead cells due to the limited number of fluorescence detection channels of the cytometer. Markers were set according to cells stained with fluorochrome-conjugated isotype control antibodies (all from BD Biosciences). For this analysis, only a very limited number of samples were available (22 for placebo and 30 for prednisolone), as many baseline PBMC samples were lost due to freezing or storage problems.

## Results

### Baseline characteristics

**Fig 1:**
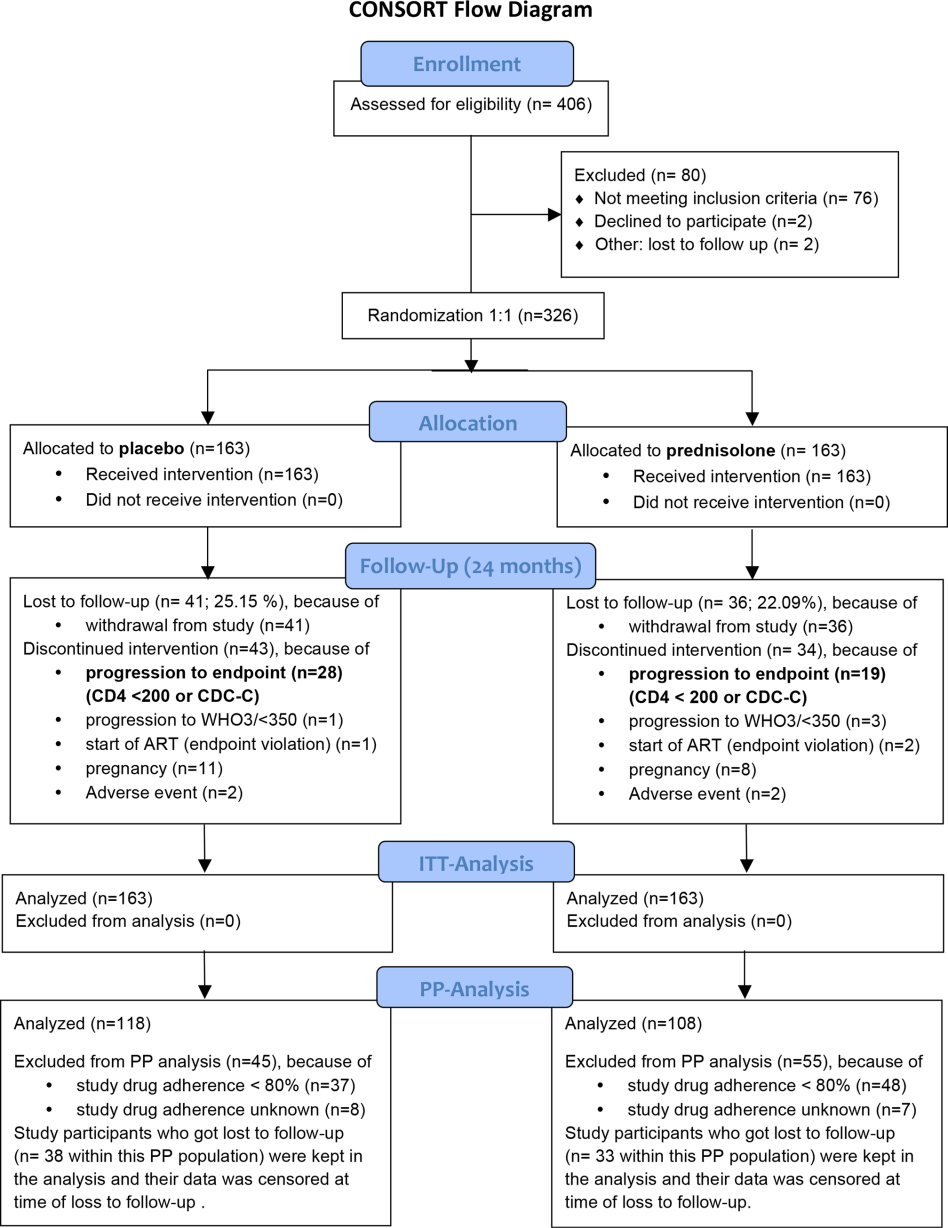
CONSORT statement 2010 flow diagram. The number of participants enrolled, randomized, allocated to study medication, followed-up and analyzed is shown. Study participants who progressed to the endpoint of the study (CD4 < 200 or CDC stage-C disease) received HAART. In some cases, study participants also received HAART when they progressed to CD4 < 350 in combination with WHO stage 3-disease. This was in accordance to the National Tanzanian treatment recommendations Update in 2008 (1 case in the placebo arm and 3 cases in the prednisolone arm). In addition, one patient in the placebo arm and 2 study participants in the prednisolone arm received HAART without fulfilling either the study endpoint or the criteria listed in the National Treatment recommendation update.

From 06/2007 to 02/2009, 406 study participants were enrolled at Bugando Medical Center (CONSORT diagram, Fig 1) in the ProCort1 study (trial register clinicaltrials.gov, NCT01299948). Eighty study participants were excluded from randomization for either not fulfilling the inclusion criteria (n = 76), or for not having signed the informed consent (n = 2), or for loss to follow-up before randomization (n = 2). The remaining 326 study participants were randomized to receive either 5 mg/day prednisolone *per os* (n = 163) or placebo (n = 163). All randomized study participants were not yet eligible for treatment with HAART according to the local Tanzanian National Treatment Guidelines (2^nd^ edition, 2005). Randomization resulted in an equal distribution of sex in both study arms (80.4.0% females in the placebo arm versus 81.0% in the prednisolone arm, p = 0.888) (Table 1). Similar distributions resulted also for age (35.27 vs. 34.09 years, p = 0.221), body-mass index (23.44 vs. 22.15, p = 0.119), and baseline HIV viral load (4.35 vs. 4.22 log10 copies/ml, p = 0.599). Study participants in the placebo arm presented at baseline with a median CD4 cell count of 530 cells/μl compared to 470 cells/μl in the prednisolone arm (p = 0.058). A total of 57.0% of the study participants randomized into the placebo arm presented with CD4 counts above 500 cells/μl compared to 44.8% in the prednisolone arm (p = 0.0351*). Female study participants presented with a significantly higher CD4/CD8-ratio than males (0.41 vs. 0.33, p = 0.0387* in the placebo arm and 0.37 vs. 0.29, p = 0.0083** in the prednisolone arm), a sex-specific difference in the immune system that has been published previously [26]. Due to differences in body weight between females and males the fix dose of 5 mg prednisolone per day resulted in a slightly higher dose per kg body weight in females compared to males (87.7 μg/kg vs 80.6 μg/kg, p = 0.0193*).

**Table 1:** Patient characteristics.

	Placebo n = 163	Prednisolone n = 163	P value* Placebo vs. Prednisolone
**Female Sex** (%)	**80.4**	**81.0**	**P = 0.888**
**Age** (years as mean with S.D.)	**35.27 ± 0.70**	**34.09 ± 0.66**	**P = 0.221**
**Body mass index (median)** [IQR 25%-75%]	**23.44 [20.44–26.11]**	**22.15 [20.55–24.70]**	**P = 0.119**
**CD4 counts (cells/μl, median)** [IQR 25%-75%]	**530 [395–659]**	**470 [397–598]**	**P = 0.058**
females	531 [394–699]	484 [398–619.5]	P = 0.0883
males	519.5 [410.3–583.3]	447 [380–530]	P = 0.2267
P value females vs males	P = 0.2638	P = 0.3436	-
**CD4/CD8 ratio (median)** [IQR 25%-75%]	**0.39 [0.30–0.54]**	**0.35 [0.26–0.54]**	**P = 0.0632**
females	0.41 [0.31–0.57]	0.37 [0.27–0.55]	P = 0.1558
males	0.33 [0.26–0.49]	0.29 [0.20–0.71]	P = 0.1350
P value females vs males	P = 0.0387*	P = 0.0083**	-
**CD4 strata** ≥ **500 cells/μl (%)**	**57.1**	**44.8**	
**CD4 strata < 500 cells/μl (%)**	**42.9**	**55.2**	**P = 0.0351**^§^
≥ 500 females	57.3	46.2	
< 500 females	42.7	53.8	P = 0.0844^§^
≥ 500 males	56.2	38.7	
< 500 males	43.8	61.3	P = 0.2101^§^
P value females vs males	P = 1.000	P = 0.5483	-
HIV viral load** LOG10 RNA copies/ml (median) [IQR 25%-75%]	**4.35 [3.60–4.81]**	**4.22 [3.75–4.95]**	**P = 0.599**
females	4.33 [3.59–4.75]	4.20 [3.75–4.92]	P = 0.5542
males	4.57 [3.68–4.98]	4.38 [4.00–5.07]	P = 0.7202
P value females vs males	P = 0.3446	P = 0.3895	-
**study drug dosage relative to body weight (μg/kg, median)** [IQR 25%-75%]	**82.0 [71.4–90.9]**	**84.7 [76.9–94.3]**	**P = 0.0535**
females	84.7 [72.5–94.3]	87.7 [77.2–98.0]	P = 0.0884
males	78.8 [70.4–87.3]	80.6 [74.6–87.7]	P = 0.3753
P value of difference females vs males	P = 0.0868	P = 0.0193*	-

*) P-values were determined using Mann-Whitney U-test if not indicated otherwise.

**) Baseline HIV viral load data available from 86 (placebo) and 80 (prednisolone) study participants. Data as LOG10 of RNA copies/ml.

§) p-value calculated from chi square test for strata ≥ 500 cells/μl (%) vs < 500 cells/μl.

### Compliance and follow-up

**Fig 2:**
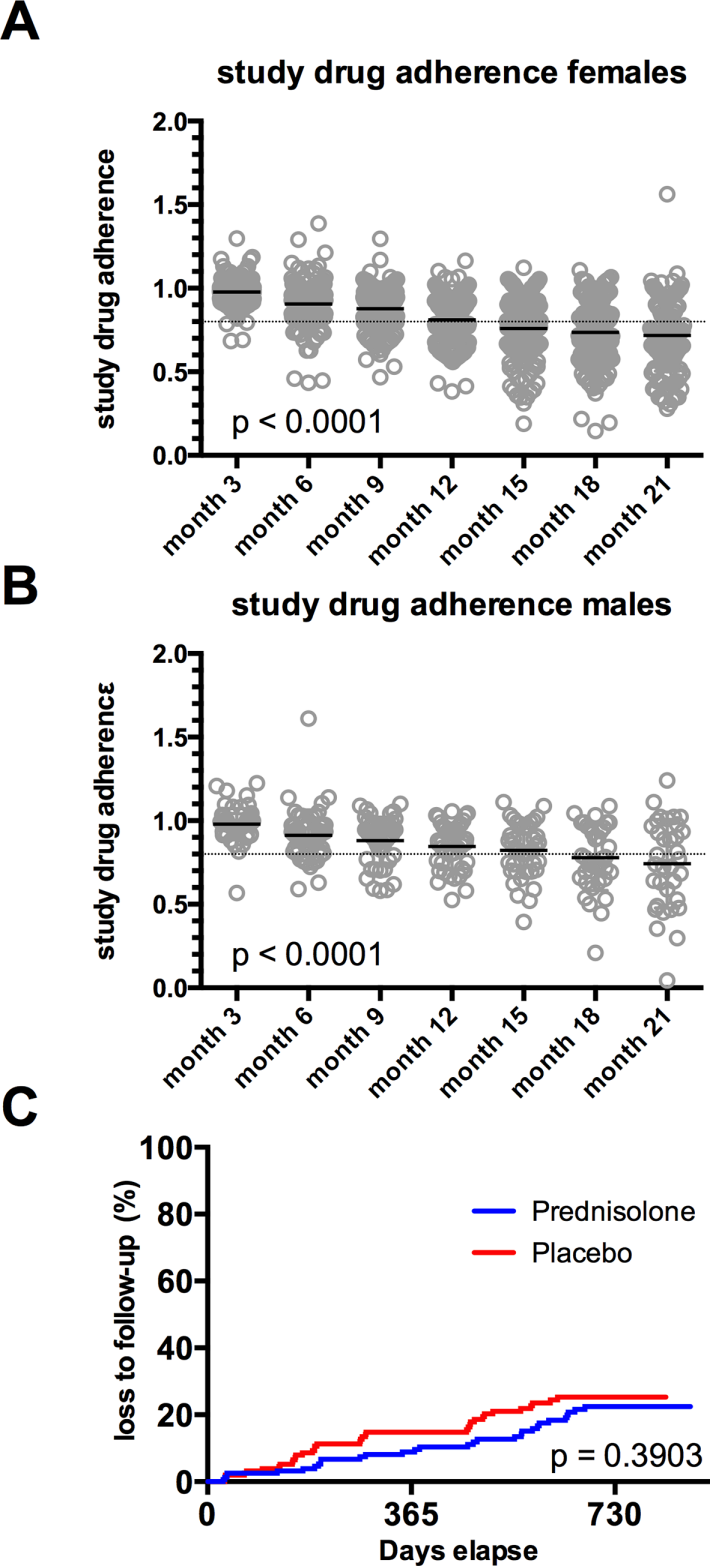
Study drug adherence and loss to follow-up. **A, B**: Study drug adherence calculated from pill counts of returned, unused study medication in female (A) and male (B) study participants. P-values were calculated by 2way ANOVA **C**: Loss to follow-up during the two-year study. P value was calculated by log-rank (Mantel-Cox) analysis.

**Fig 3:**
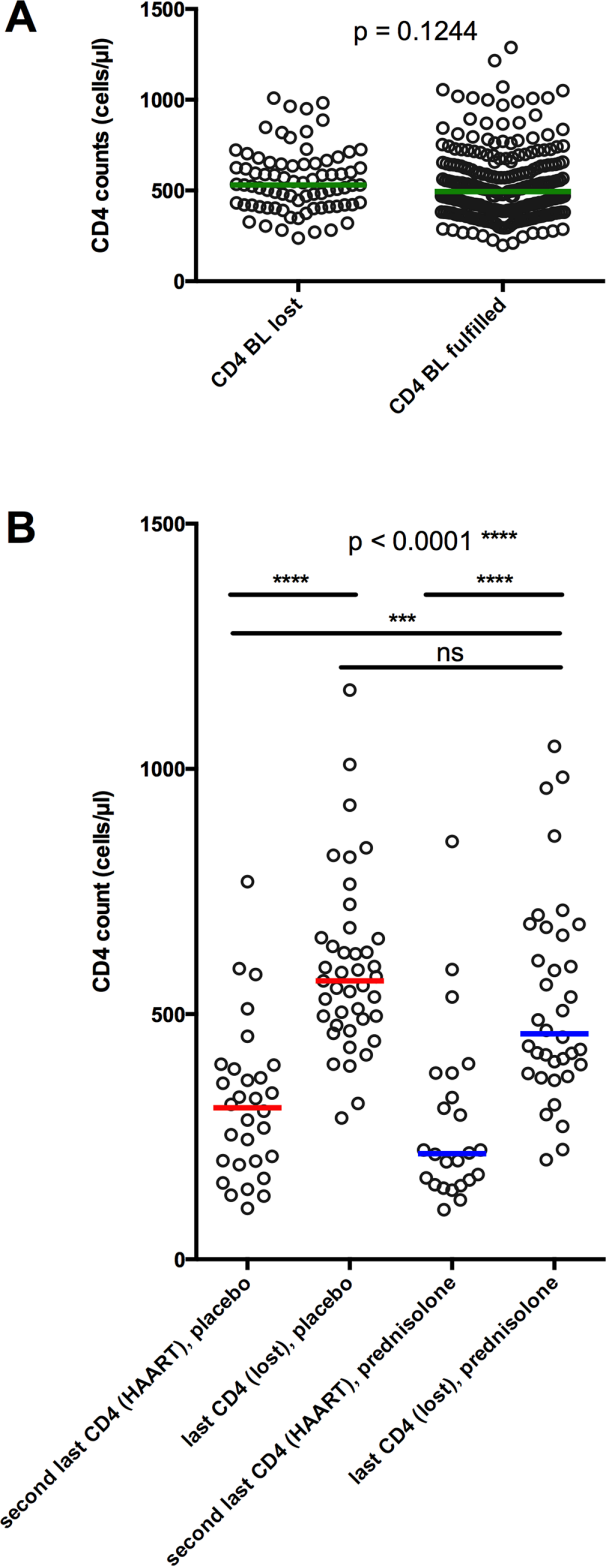
CD4 counts of study participants with loss of follow-up. **A:** Baseline (BL) CD4 counts from study participants who were later lost to follow up (lost) and who fulfilled (fulfilled) the study. P value was calculated by Mann-Whitney test. **B:** CD4 counts prior to loss to follow up (loss) or to progression to the primary endpoint (HAART). Red bars (placebo) and blue bars (prednisolone) represent medians. P values were calculated by Kruskal-Wallis test with multiple comparisons.

Compliance with study regimens was estimated by the amount of unused and returned study drug. Overall compliance was similar in both groups (data not shown) and no difference in compliance was observed between female and male study participants (Fig 2A and 2B). Compliance declined over time from a median of 98.03% at month 3 to 71.58% at month 21. Of the 326 study participants who underwent randomization, 36 study participants (22.1%) in the prednisolone group and 41 study participants (25.1%) in the placebo group were lost to follow-up during the two-year study period (difference p = 0.3903) (Fig 2C). The majority of them did not attend anymore the visits of the study despite multiple efforts to remind them by telephone. Others came from rural areas and moved back to their home villages during the study, which also resulted in the discontinuation of study participation. As depicted in Fig 3A, study participants who were later lost to follow-up did not present with lower initial CD4 counts at baseline than study participants who fulfilled the study. Participants who got lost to follow-up also presented with significantly higher CD4 counts at their last visit than study participants who were at their next visit diagnosed with AIDS and who received HAART (Fig 3B, p < 0.0001). We therefore conclude that loss to follow-up in the study was probably not related to HIV disease progression.

### Effects of prednisolone on clinical disease progression

**Fig 4:**
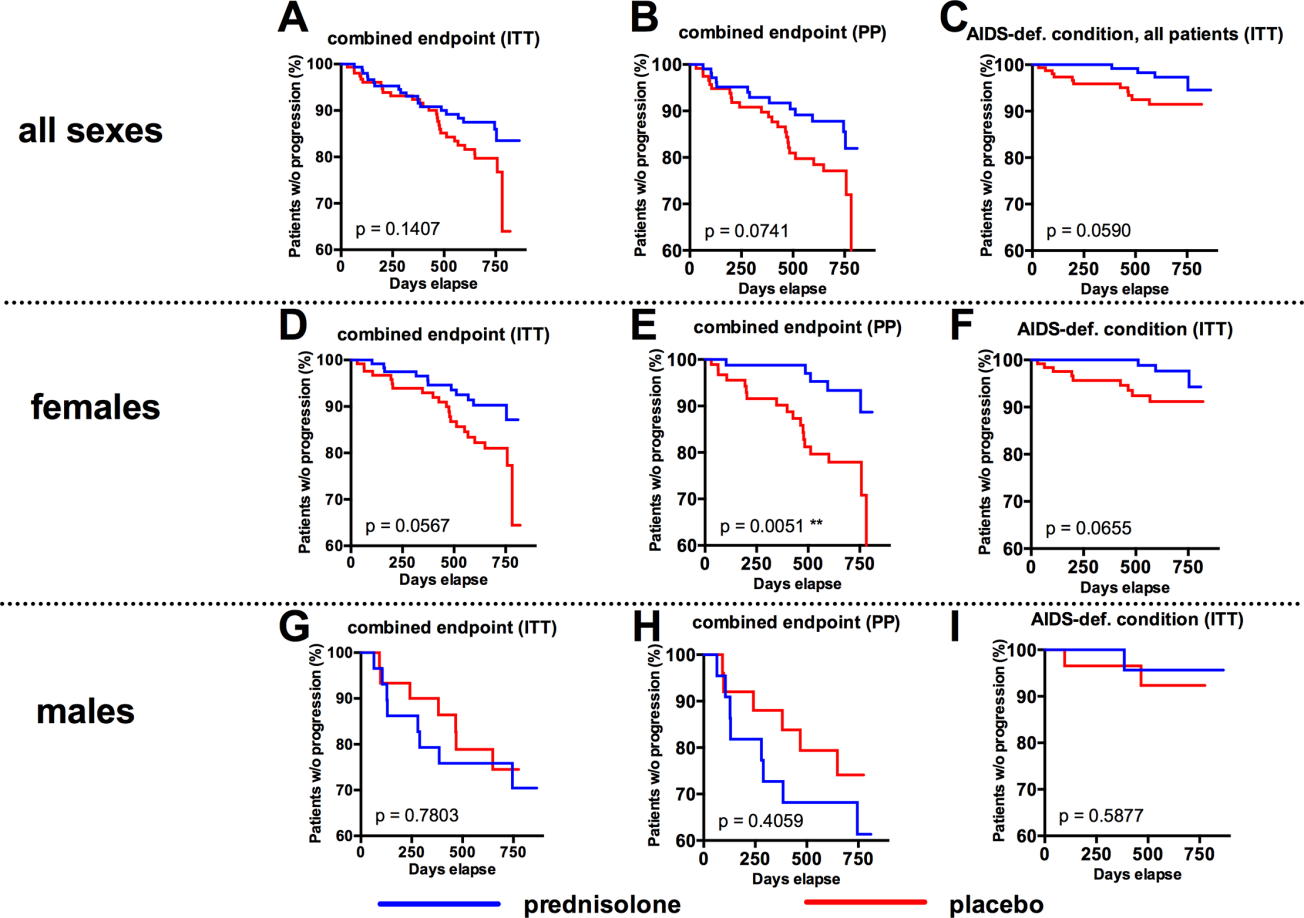
Effects of prednisolone on HIV disease progression: Primary Study Endpoint. Kaplan-Meyer estimate of progression to combined study endpoint (onset of CDC stage-C-condition or drop of CD4 counts below 200) within the intent-to-treat (ITT) population (**A**) or the per protocol (PP) population (**B**). **C**: Progression to AIDS-defining condition. All study participants who received HAART, but did not reach the study endpoint were censored for the KM analysis. **D-F** and **G-I**: Post-hoc analyses for female study participants (D-F) and for male study participants (H-I) for progression to combined endpoint within the ITT population (D, G), progression to combined endpoint within the PP population (E, H), and progression to AIDS-defining condition (F, I). A-I: P values were calculated by log-rank (Mantel-Cox) analysis.

The primary endpoint of the study was progression to CDC stages A3, B3, C or death. This endpoint was reached if CD4 counts dropped below 200 cell/μl (CDC stage 3) or if an AIDS-defining condition occurred (CDC stage C), or if the patient died. Study participants who reached this combined endpoint received HAART. During the study, no patient died and 19 study participants in the prednisolone arm and 28 study participants in the placebo arm reached the primary study endpoint (Table 2). These numbers were subjected as intent-to-treat (ITT) to a Kaplan-Meyer analysis (Fig 4A). The difference was statistically insignificant (log-rank test p = 0.1407).

**Table 2:** Clinical endpoints and other reasons for initiating HAART.

Reasons for initiating HAART			Prednisolone	Placebo
**Clinical study endpoints**				
	**AIDS-defining conditions**	**Overall**	**4**	**11**
		Candidiasis oesophageal	0	1
		Cervix carcinoma	0	1
		Kaposi sarcoma	0	1
		M. tuberculosis disease	3	3
		Toxoplasmosis of the brain	0	1
		P. jirovecii pneumonia	1	1
		Pneumonia recurrent	0	2
		Wasting syndrome	0	1
	**CD4 < 200**		**15**	17*
	**Death**		**0**	**0**
**total reaching clinical study endpoint**			**19**	**28**
**Non-endpoints**^§§^				
	**CD4 < 350 and WHO stage 3**^§^	**Overall**	**5**	**2**
		candidiasis, oropharyngeal	1	0
		cough, fever, weight loss	1	0
		fever, prolonged	0	1
		tonsillitis, pneumonia	1	0
	**other reasons**^#^	candidiasis, oral, CD4 of 380 cells/μl	1	0
		asymptomatic, low CD4 count, but above 200 (203 and 201)	1	1
**total receiving HAART**			**24**	**30**

*) one patient receiving placebo developed an AIDS-defining condition with CD4 counts < 200. In this table this patient was only listed under AIDS-defining conditions, and not also under the CD4 criterion.

§) During the study, the National Treatment guidelines in Tanzania were updated in 2009. Since then, the initiation of HAART was indicated at WHO stage 4, or at CD4 counts < 350 cells/μl if a (non-AIDS-defining) opportunistic infection (WHO stage 3) is present. Due to difficulties in drug supply this update was fully implemented at out study site only since 2011. A total of 4 study participants fulfilled this condition and discontinued participation in the study and received HAART.

#) In one case the decision to initiate HAART was not in accordance with the study protocol or the national guidelines, but was done at the discretion of the treating clinician. Before initiation of HAART at study month 4, and with a CD4 count of 380 cells/μl and a non-AIDS-defining condition (oral candidiasis), the patient presented with stable CD4 counts of 723 cells/μl (baseline), 754 cells/μl (month 1), 524 cells/μl (month 2), and 812 cells/μl (at month 3). CD4 counts continued to be stable immediately after initiation of HAART (486 cells/μl at week 6 and 714 cells/μl at week 10). In two cases, HAART was initiated in asymptomatic infection at 203 (prednisolone) and 201 (placebo) cells/μl.

§§) These study participants were censored in the Kaplan-Meyer analysis.

**Fig 5:**
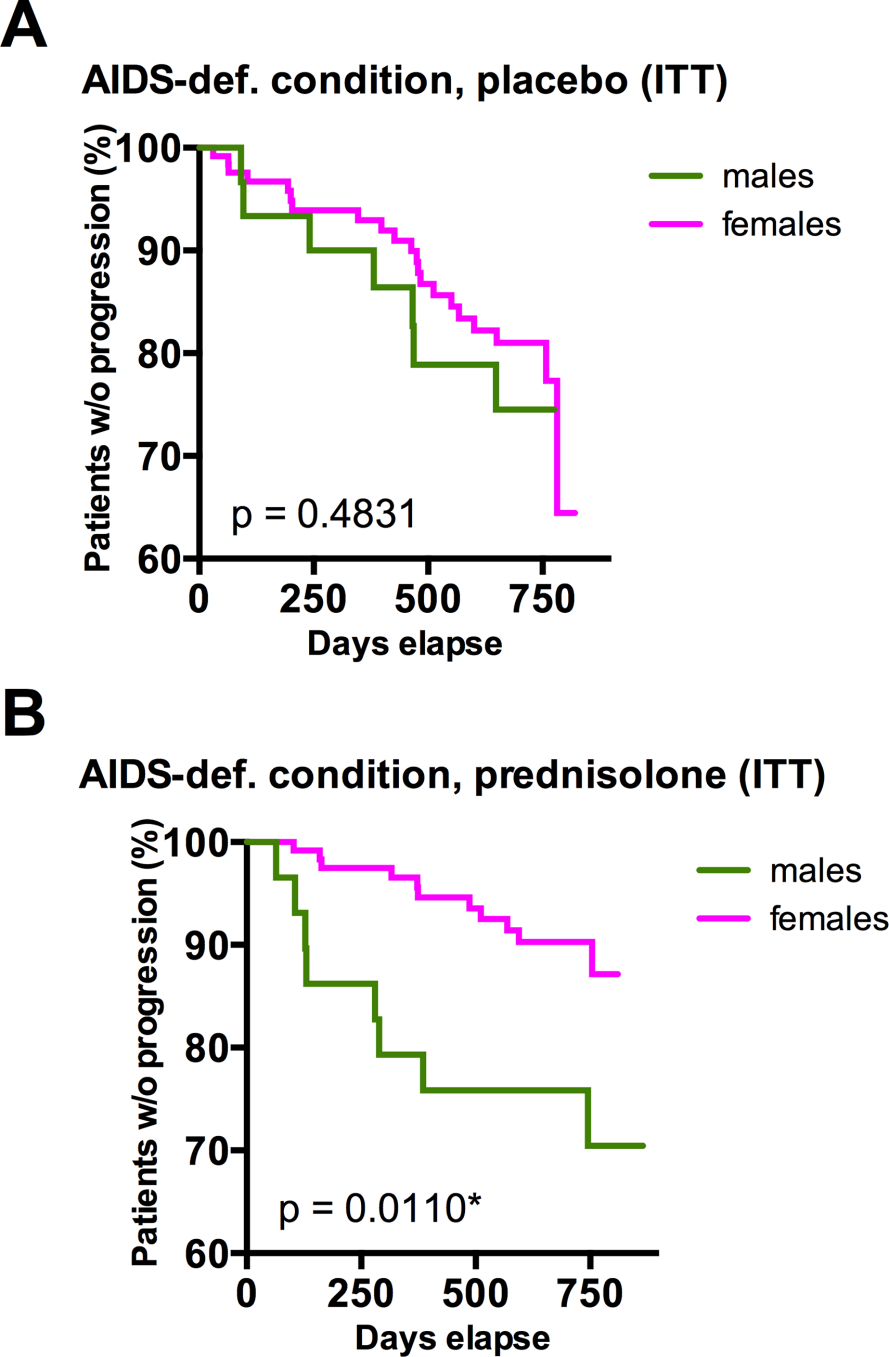
Disease progression in male and female study participants. Kaplan-Meyer estimate of progression to combined study endpoint (onset of CDC stage-C-condition or drop of CD4 counts below 200) within the intent-to-treat (ITT) population of study participants treated with placebo (**A**) or prednisolone (**B**) stratified by sex. P values were calculated by log-rank (Mantel-Cox) analysis.

The protocol defined that the minimum average adherence to the study drug had to be greater than 80%. In a per protocol (PP) analysis in which we excluded study participants with an average study drug adherence less than 80%, 13 (prednisolone) versus 24 (placebo) participants (from a total of 108 and 118, respectively) progressed to the primary end point (Fig 4B, log-rank test p = 0.0741). The PP analysis that we performed in this study is technically a “modified ITT analysis”, as we also included the data from participants who were lost to follow-up in order to compensate for the high drop-out rate in our study. If we perform a strict PP analysis, in which participants who got lost to follow-up are excluded, the number of analyzed individuals drops to 82 in the prednisolone arm versus 88 in the placebo arm, and the log-rank test result increases to p = 0.0917 (graph not shown). A post-hoc analysis for progression to an AIDS-defining condition resulted in 4 (prednisolone) versus 11 (placebo) cases (Fig 4C, log-rank test p = 0.0575). A post-hoc analysis stratified for female sex resulted in 11 (prednisolone) versus 21 (placebo) cases reaching the primary endpoint in the ITT analysis (log-rank test p = 0.0567, Fig 4D) and 5 (prednisolone) versus 18 (placebo) cases in the PP analysis (log-rank test p = 0.0051**, Fig 4E. (The PP analysis excluding participants lost to follow-up results in a p = 0.0078**, graph not shown). No differences in disease progression between the placebo- and prednisolone-arm were observed in men (Fig 4G–4I).

As described above, the PP-population in this study is defined as the population with an *average* study drug adherence of more than 80%. If we do an analysis restricted to study participants who *never* dropped below the required 80% minimum drug adherence, we get a similar picture as in the PP analysis: No differences in disease progression in men, but a significant attenuation of disease progression by prednisolone in women (1 in the prednisolone arm versus 8 in the placebo arm, from a total of 119 and 121, respectively, log-rank test p = 0.0235*, graph not shown).

Despite the relatively low number of male study participants (about 30 in each arm), the observed interaction by sex was statistically significant: In the prednisolone arm, females progressed significantly slower to the primary end point than males (Fig 5B, log-rank-test p = 0.0110*), whereas no differences in disease progression between females and males were observed in the placebo arm (Fig 5A, log-rank-test p = 0.4831).

**Fig 6:**
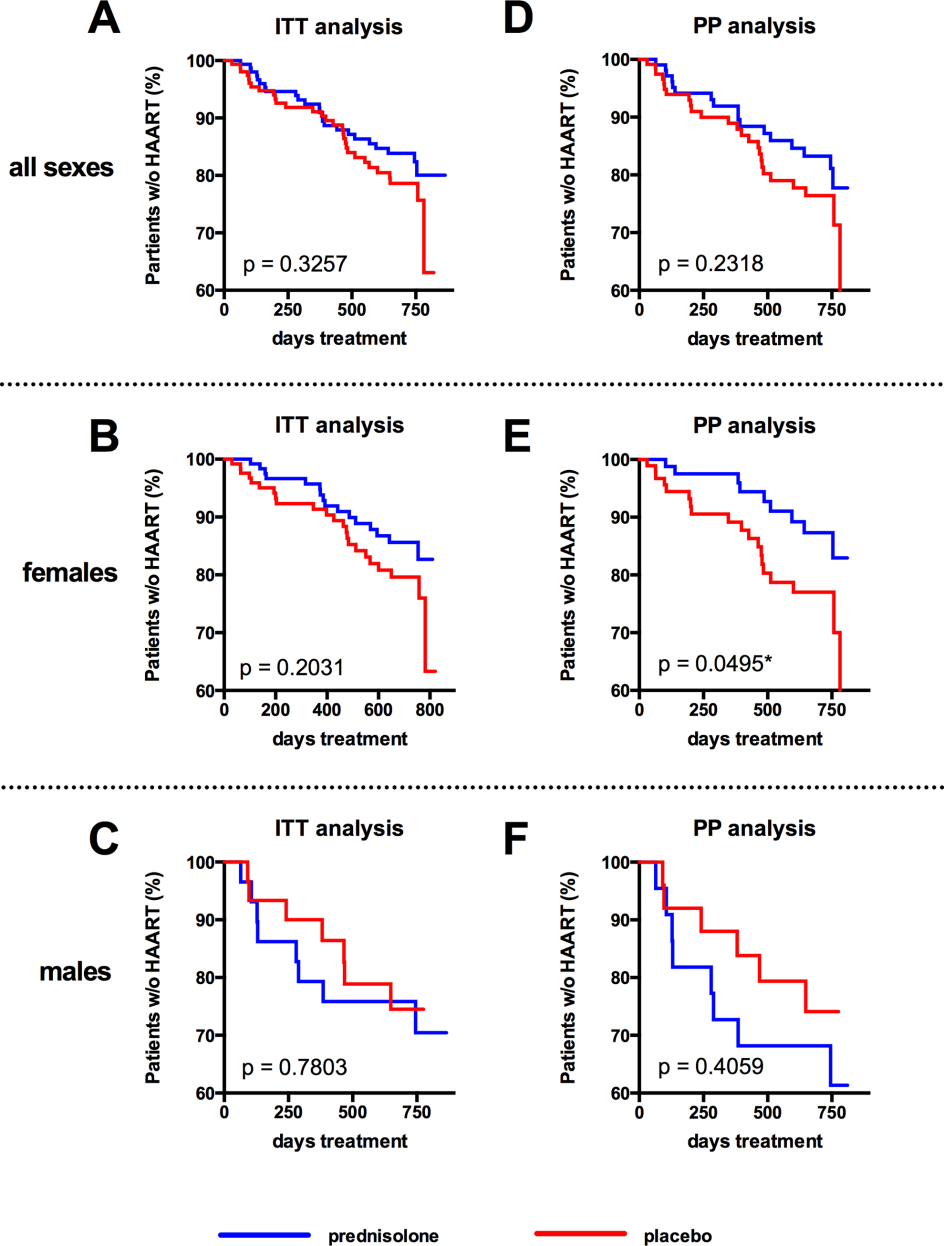
Progression to HAART as treated. Kaplan-Meyer estimate of progression to HAART as treated within the intent-to-treat (ITT) population (**A**) or the per protocol (PP) population (**B**). Separate analyses for female study participants (B, E) and for male study participants (C, F) for progression to HART as treated. P values were calculated by log-rank (Mantel-Cox) analysis.

**Fig 7:**
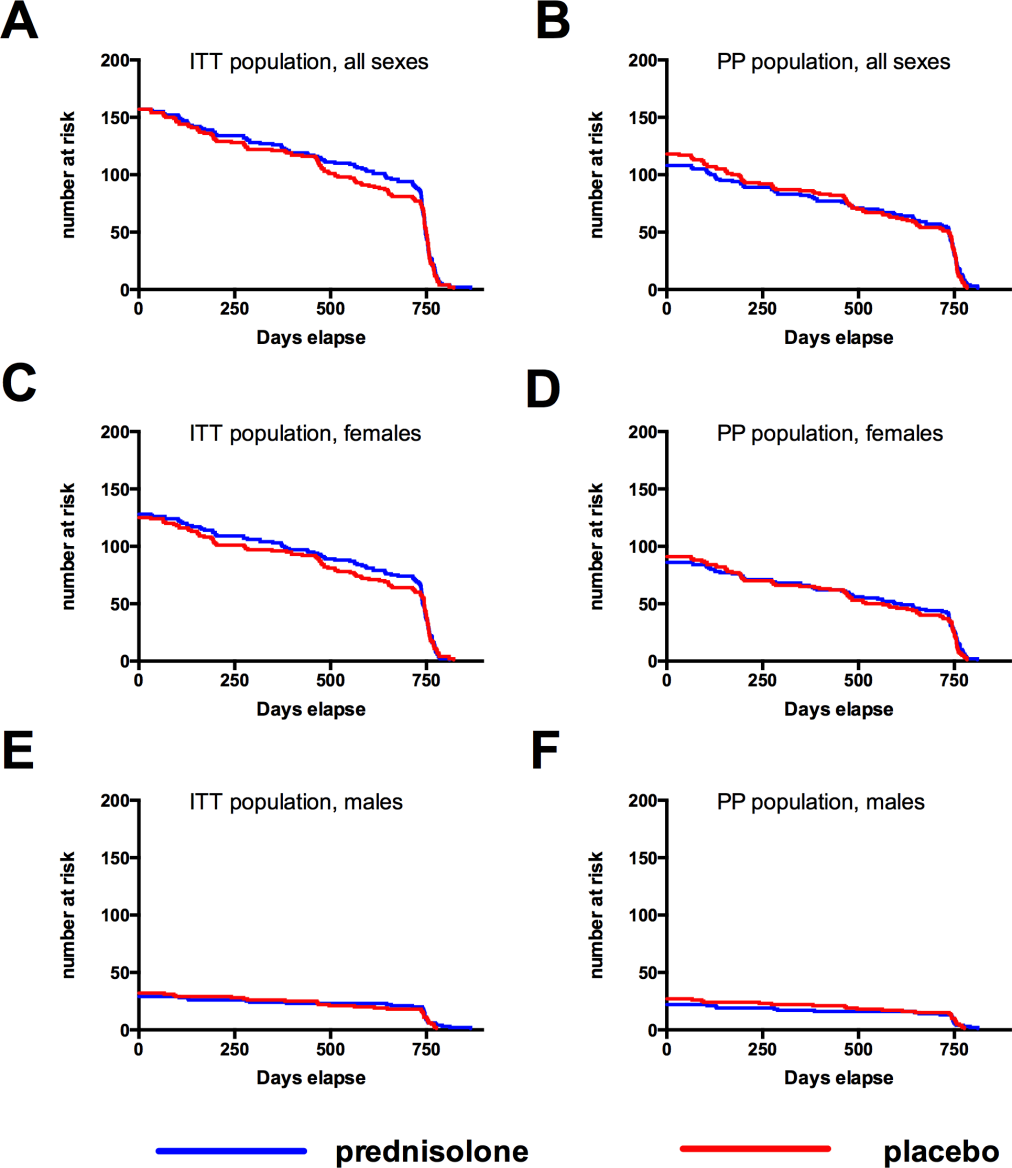
Study participants at risk. Number of study participants at risk within the intent-to-treat (ITT) population (**A**) or the per protocol (PP) population (**B**). Separate analyses for female study participants (C, D) and for male study participants (E, F).

In addition to the 19 (prednisolone) and 28 (placebo) study participants who received HAART for fulfilling the criteria of the primary study endpoint (CD4 < 200 or CDC stage-C-disease), 7 more study participants (5 in the prednisolone group and 2 in the placebo group) progressed to HAART (Table 2). Four of them (3 in the prednisolone group and 1 in the placebo group) presented with CD4<350 in combination with a WHO-stage 3-disease. According to an update in the Tanzanian National treatment guidelines in 2009 that was released during the study, this was an indication for start of antiretroviral treatment. The other three (2 in the prednisolone group and 1 in the placebo group) started HAART by discretion of the treating physician, but without fulfilling either the study endpoint criteria nor the criteria listed in the National Treatment Guideline Update from 2009. The total number of participants receiving HAART in the study was therefore 24 (prednisolone) versus 30 (placebo) (Fig 6A–6C shows the ITT analysis for all sexes (A), females (B) and males (C); no differences were detected). In a PP analysis, females treated with prednisolone still progressed significantly slower to HAART than females receiving placebo (log-rank test p = 0.0495*, Fig 6E), whereas no differences were observed in men (log-rank test p = 0.4059, Fig 6F). Fig 7 shows the number at risk for the ITT and PP populations of the Kaplan-Meyer analyses depicted in the Figs 4 and 6 for all sexes (Fig 7A), females (Fig 7B), and males (Fig 7C).

### Effects of prednisolone on CD4 counts

**Fig 8:**
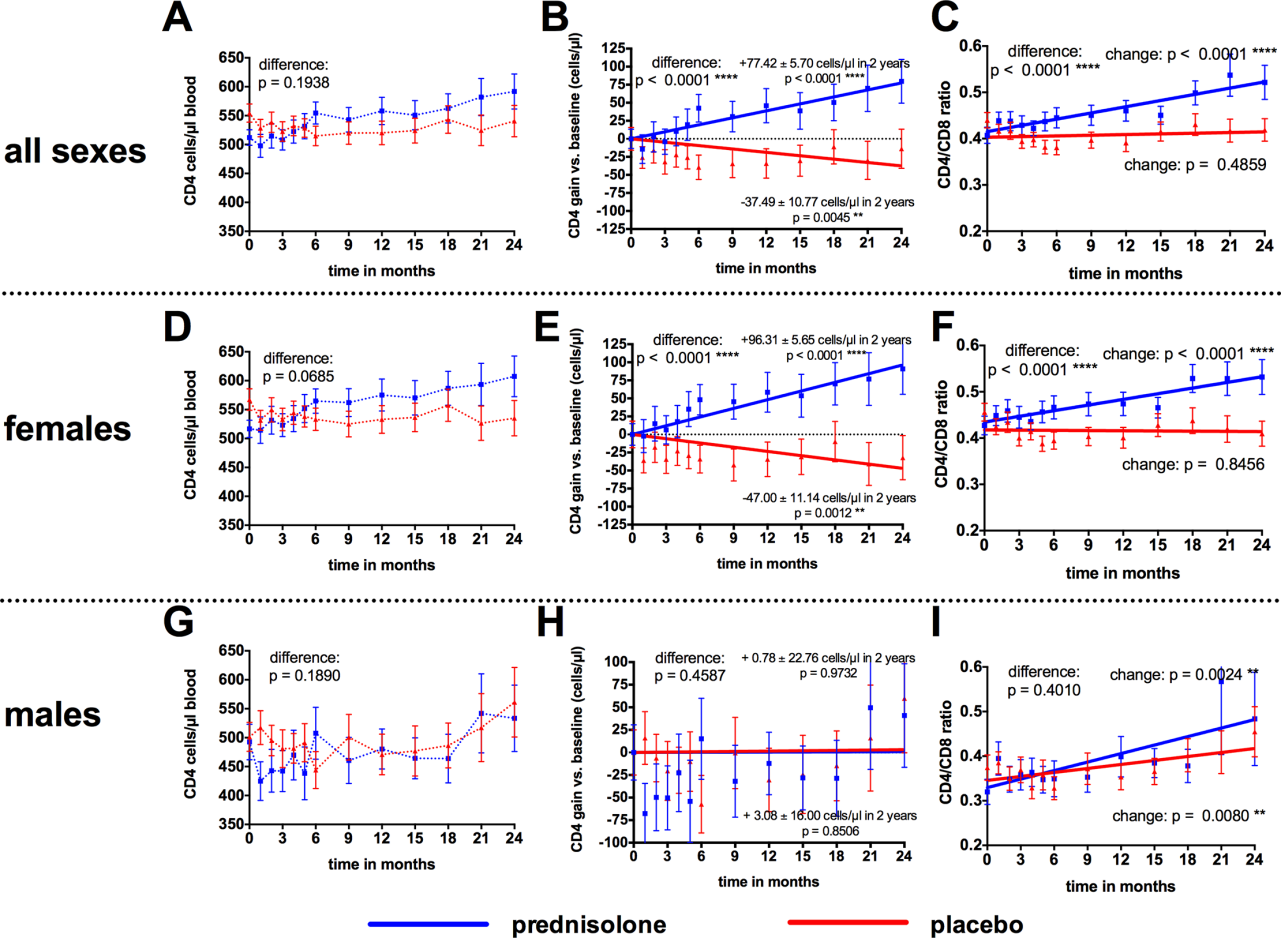
Effects of prednisolone on CD4 counts and CD4/CD8 ratio. **A**: Mean CD4 ± S.D. P-values were determined by 2way ANOVA. **B**: Mean CD4 changes ± S.D. relative to baseline and linear regression of the data. 2-year gains/losses were calculated from slope of linear regression. **C**: Mean ± S.D. CD4/CD8-ratio and linear regression. B, C: P values on the right indicate non-zero hypotheses for the slope. P-value on the left for difference between the two data sets was calculated by 2way ANOVA. **D-F** and **G-I**: Post-hoc analyses for female study participants (D-F) and for male study participants (H-I) for absolute CD4 counts (D, G), CD4 relative CD4 changes (E, H), and CD4/CD8 ratio (F, I).

Prednisolone treatment had a stabilizing effect on CD4 counts, with an average increase of +77.42 (± 85.70) cells/μl for prednisolone during the 2-year study period calculated by linear regression (p < 0.0001) (Fig 8B). In contrast, study participants treated with placebo experienced a significant decline of CD4 counts by -37.49 (± 10.77) cells/μl (p = 0.0005). Differences in CD4 changes between the two treatment groups were statistically significant (p < 0.0001). The difference in absolute CD4 counts between the two treatment arms did not reach statistical significance (p = 0.1938) (Fig 8A). Prednisolone treatment also significantly improved the CD4/CD8 ratio of the study participants compared to placebo (p < 0.0001) (Fig 8C). These positive effects of prednisolone on CD4 cell counts and CD4/CD8 ratio were even more pronounced when analyzing female study participants separately. In contrast, no effects were observed in male study participants (Fig 8D–8F for females and Fig 8G–8I for males).

### Effects of prednisolone on immune activation and viral load

**Fig 9:**
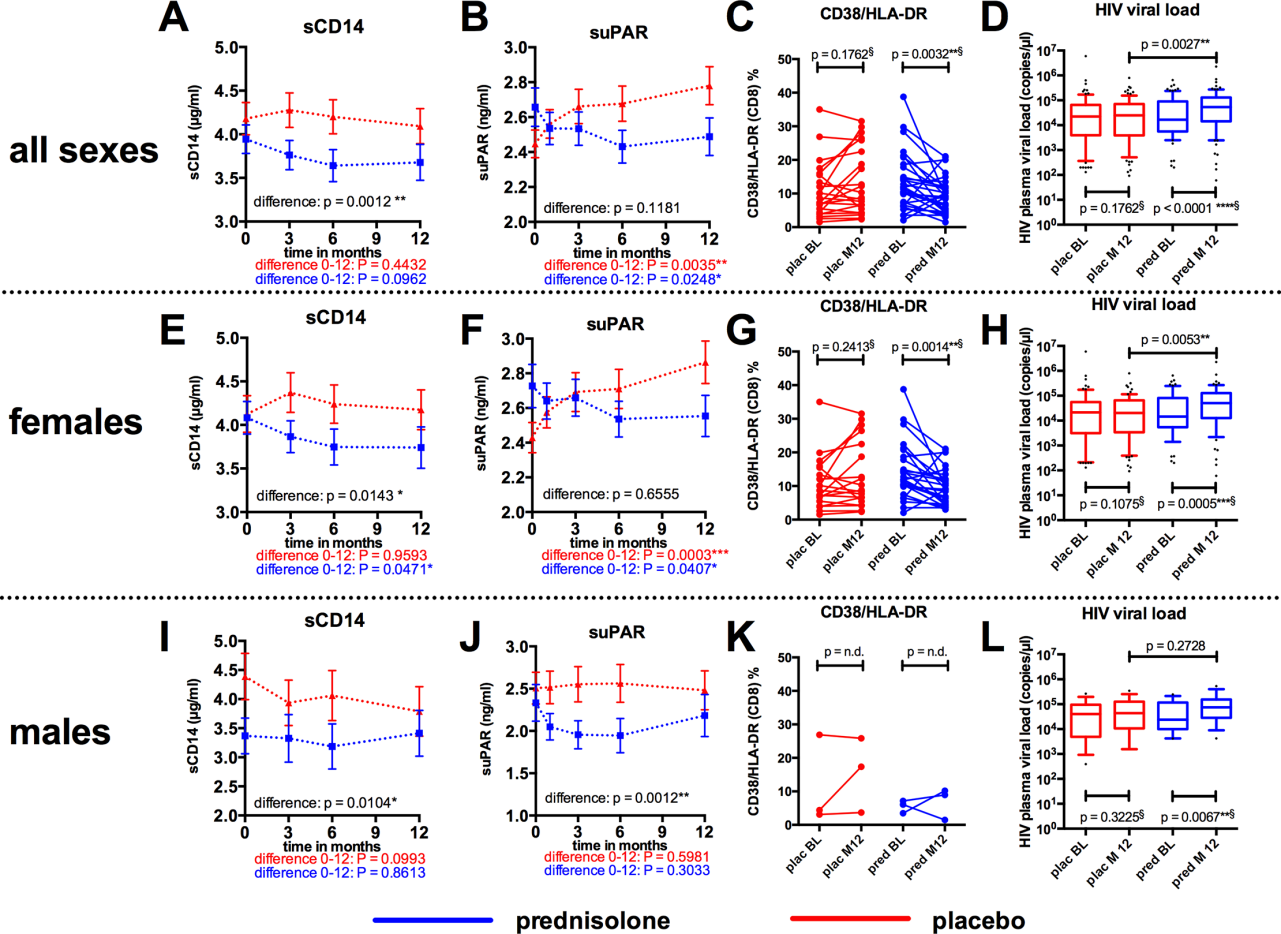
Effects of prednisolone on immune activation and HIV viral load. **A, E, I:** Concentration of sCD14 was determined by ELISA from n = 134 (placebo) and n = 136 (prednisolone) available plasma samples collected at baseline, 3, 6 and 12 months. A: all sexes, E: females (n_Plac_ = 106, n_Pred_ = 110), I: males (n_Plac_ = 28, n_Pred_ = 26). **B, F, J:** Concentration of sUPAR was determined by ELISA from n = 122 (placebo) and n = 124 (prednisolone) available plasma samples collected at baseline, 3, 6 and 12 months. B: all sexes, F: females (n_Plac_ = 95, n_Pred_ = 102), J: males (n_Plac_ = 27, n_Pred_ = 22). A, B, E, F, I, J: Data as means ± S.D. P-values were determined by 2-way ANOVA (difference between the two treatments over the whole time period) or by Wilcoxon matched-pairs signed test (changes between Baseline and month 12). **C, G, K:** CD38/HLA-DR expression was determined by flow cytometry from n = 22 (placebo) and n = 30 (prednisolone) available frozen PBMC samples collected at baseline and 12 months. C: all sexes, G: females (n_Plac_ = 19, n_Pred_ = 27), K: males (n_Plac_ = 3, n_Pred_ = 3). P-values were determined by Wilcoxon matched-pairs signed test (changes between Baseline and month 12). **D, H, L:** HIV viral load was determined from n = 86 (placebo) and n = 80 (prednisolone) available plasma pairs at baseline and month 12. D: all sexes, H: females (n_Plac_ = 70, n_Pred_ = 66), L: males (n_Plac_ = 16, n_Pred_ = 14). P-values were determined by Wilcoxon matched-pairs signed test (changes between Baseline and month 12) and by Mann-Whitney test (comparison of month 12 placebo versus month 12 prednisolone).

Treatment with prednisolone resulted in a moderate but significant reduction of immune activation (Fig 9). Soluble CD14 (sCD14) as well as soluble urokinase-type plasminogen activator receptor (suPAR), two plasma markers that have been described to correlate with HIV disease progression [5, 27], were significantly decreased following prednisolone treatment compared to placebo (Fig 9A and 9B). Coexpression of CD38 and HLA-DR on CD8-positive T-cells, a set of markers that have previously been described as a very reliable progression marker for HIV disease [2, 3, 7, 8], was also significantly reduced in the prednisolone arm (Fig 9C). The number of samples that we could use for this analysis was limited due to freezing problems of the PBMC during storage and shipment (n = 22 for placebo versus n = 30 for prednisolone). We also determined HIV viral load from 166 available plasma sample pairs from baseline and month 12. As depicted in Fig 9D, prednisolone treatment caused a statistically significant increase in HIV viral load at month 12 compared to baseline by a factor of 3.2 (median 1.65 x 10^4^ copies/ml at baseline compared to median 5.28 x 10^4^ copies/ml at month 12, p < 0.0001, Wilcoxon matched-pairs signed rank test).

In order to detect any effects related to sex [28], we performed a post-hoc analysis and analyzed the outcome for female and male study participants separately. A reduction of immune activation was most pronounced in females (Fig 9E–9G), whereas only little effects were observed in men (Fig 9I–9K). In contrast to immune activation, the enhancing effects of prednisolone on HIV viral load could be detected both in females and in males (Fig 9H and 9L).

### Adverse events

The rate of adverse events, including typical side effects of higher-dose prednisolone, was not elevated during prednisolone treatment (Table 3). However, there are long-term toxicities of prednisolone that were not evaluated during this study, such as osteoporosis. An interesting observation is the significant reduction of malaria cases in prednisolone-treated study participants compared with placebo-treated study participants (33 cases versus 59 cases, p = 0.0083**, Table 3), which merits further investigation in future trials. As we did not systematically screen for malaria infections in this trial, this observation might as well be an effect of prednisolone-mediated masking of malaria-triggered fever resulting in an underreporting of malaria cases in the prednisolone arm. Also the condition “pain” was reported less frequently under prednisolone treatment than under placebo treatment (60 versus 88 cases, p = 0.0237*, Table 3). We observed more skin disorders such as rash or tinea under prednisolone treatment (79 versus 56 cases, p = 0.0540, Table 3). The body–mass-index (BMI) increased in both study arms during the 2-year study time by about 1.5 BMI units and no differences between study participants receiving prednisolone or placebo were observed (data not shown). We also measured glucose levels in urine and no changes over time were observed in either treatment group (data not shown).

**Table 3:** Adverse Events.

	Adverse event (AE)	Prednisolone		Placebo		P Value^§^	
		cases	events	cases	events	cases	Events^§§^
general	Any adverse event	144	669	143	711	1.0000	n.d.
	Any serious AE	15	16	14	15	1.0000	n.d.
	Any grade 3/4 AE	8	9	8	10	1.0000	n.d.
	grade 3 event	7	8	8	9	1.0000	n.d.
	grade 4 event	1	1	1	1	1.0000	n.d.
infections	Herpes	15	16	12	14	0.6884	0.8546
	Malaria	30	33	41	59	0.1794	0.0083*
	Pneumonia	26	28	19	22	0.3354	0.4767
	Respiratory tract	75	122	68	113	0.5031	0.5904
	Tonsillitis	6	8	15	16	0.0689	0.1518
	Tuberculosis	3	3	3	3	1.0000	0.6829
	Urinary tract	24	30	24	28	1.0000	0.8948
	Candidiasis	22	32	28	38	0.4424	0.5465
gastrointestinal symptoms	Abdominal pain	33	38	31	41	0.8892	0.8202
	Diarrhoe	24	32	34	41	0.1922	0.3446
	Epigastric pain	8	12	12	13	0.4896	1.0000
	Nausea	10	12	8	8	0.8092	0.5012
	Peptic ulcer	2	3	6	6	0.2830	0.5045
	Vomiting	4	4	3	3	1.0000	1.0000
cancers	Kaposi sarcoma	0	0	1	1	1.0000	1.0000
common side effects of prednisolone	Hyperglycemia	1	1	1	1	1.0000	0.4794
	Fluid retention	0	0	1	1	1.0000	0.9855
	Skin disorders	50	79	39	56	0.2137	0.0540
	Hypertension	0	0	0	0	n.a.	n.a.
	Pain	37	60	58	88	0.0146*	0.0237*
others	Fever	45	54	47	65	0.9021	0.3519
	Neurol. disorders	5	5	5	5	1.0000	0.7515
	Other^#^	71	99	63	88	0.4308	0.2479

#: conjunctivitis, otitis media, prolonged menstruation, dental pain, flu-like symptoms, worm infections, dental pain, dizziness, numbness etc.

§) P from Χ^2^ calculation

§§) the maximum number of reported events per condition is 1956 (163 study participants x 12 visits). This number was used as the basis for the Χ^2^ calculation for number of events.

*) statistically significant with p < 0.05.

## Discussion

The primary endpoint of the study did not reveal any differences in disease progression towards onset of AIDS (defined as a drop of CD4 counts below 200 cells/ml or the onset of a clinical AIDS-defining condition). However, prednisolone decreased immune activation and increased CD4 counts. Prednisolone treatment also caused an increase in HIV viral load. A per protocol analysis stratifying for sex (post-hoc) showed a significant attenuation of disease progression in female study participants treated with prednisolone compared to placebo.

The study has potential limitations. First, the primary endpoint of the study, “progression to AIDS”, contains both a laboratory surrogate marker (CD4 counts), as well as a purely clinical endpoint (onset of an AIDS-defining condition). The majority of study participants who progressed to this combined endpoint met the CD4 criterion (15 versus 17 for prednisolone and placebo, respectively), whereas only a much smaller fraction of study participants reached the clinical endpoint “onset of AIDS-defining condition” (4 versus 11), and differences in this purely clinical endpoint did not quite reach statistical significance (p = 0.0575). Since we cannot exclude that prednisolone-mediated changes in CD4 counts could also be related to a different distribution of cells—instead of an increase in total numbers—the answer to the question whether prednisolone treatment in HIV infection indeed improved health must be given with caution. We suggest, however, that the observation of a long-lasting increase in CD4 counts over the entire two-year treatment period argues against a mere redistribution effect and in favor of a true increase in CD4 numbers.

Second, the study has a two-year drop-out rate of about 24%, which is substantially higher than the primary endpoint event rate of about 14% and therefore may compromise the Kaplan-Meier-analysis. This drop-out rate (which is equivalent to an annual 12% loss-to follow-up) is comparable to the loss-to-follow-up rates reported in many other clinical trials on HIV drug adherence in Sub-Saharan Africa [29]. As the drop-out rate in the prednisolone arm was similar to the drop-out rate in the placebo arm (22.09% versus 25.15%, respectively) we suggest that there is reason to assume that the dropout rate may not have had a big distorting effect on the results of the trial and we found no evidence that the drop-outs were related to HIV infection.

Third, stratification according to sex was a post-hoc analysis, and only in this post-hoc analysis we observed a statistically significant benefit for prednisolone in attenuation of disease progression in female study participants. By definition randomization is lost in post-hoc analyses and the data must thus be considered with caution. Differences in outcome between male and female study participants could be attributed to sex-specific differences in the immune system as well as to different pharmacokinetics and pharmacodynamics of prednisolone in females and males. Sex-specific differences in the immune responses have been known for decades and have also been observed in HIV infection (reviewed in [28]), and one of these differences were also directly observable in the baseline characteristics of this study, showing a higher CD4/CD8 ratio in females, in accordance to what has been reported by other groups [26]. In particular, HIV-associated immune activation was found to be higher in females than in men [30], indicating that immune intervention therapies might also have different results in females and males. Pharmacokinetic reasons may also account for the observed differences. A study on prednisolone pharmacokinetics in relation to sex and race found a higher AUC in females versus males, as well as both a higher clearance and a higher volume of distribution in males versus females [31]. These differences may explain why the fix, relatively low dose of 5 mg prednisolone given in the study might have shown less activity in males. Moreover, when adjusted to bodyweight, females received a 9% higher dose of prednisolone, which may also have contributed to the observed differences between females and males.

Fourth, the data on immune activation and HIV viral load derive only from some time points of the study and only from a limited number of samples, either due to limitations in funding and/or lack of availability of samples. For example, HIV viral load data was determined from all study participants from whom we had both baseline and month 12 plasma samples available, corresponding to a total of 166 study participants (51% of the study population). This selection has the bias of excluding, a priori, all study participants from this analysis who either fulfilled the study before month 12 or who were lost to follow-up before month 12 (which combined account for about 25% of the study population). Changes in HIV viral load detected in the month 12-samples compared to baseline may therefore not necessarily represent viral load changes in the rest of the study population.

The mechanism of the observed increase in HIV viral load is unclear. HIV viral load is influenced by a variety of parameters and some of them have opposite effects on HIV replication, including CD4 T cell activation (which is a prerequisite for productive infection and which prednisolone would probably counteract) and CTL activity (which plays a major role in immunological control of HIV replication, which is potentially inhibited by prednisolone treatment). Low-dose prednisolone as investigated in this study has obviously shifted this balance towards enhanced virus replication. Several previous clinical trials have studied the effects of higher-dose corticosteroids on HIV infection and HIV infection: In a prospective, uncontrolled clinical trial, Andrieu et al. found an increase in CD4 counts by 119 cells/μl within one year in antiretroviral-untreated HIV infection. Prednisolone was given at 0.3–0.5 mg/kg body weight (about 4–6 times higher than in our study) and no changes in HIV viral load were detected [22, 23]. Elliott et al. studied the effects of prednisolone on HIV-associated pleural tuberculosis in 197 antiretroviral-untreated study participants in a double-blind, randomized placebo-controlled clinical trial. Prednisolone was given at a dosage of 50 mg per day (which is ten times higher than in our study) for two weeks and then tapered down to 15 mg in the following 6 weeks before it was stopped. No changes in HIV viral load or CD4 counts have been detected [32]. Wallis et al. studied the effects of prednisone on antiretroviral-treated HIV infection in a randomized, placebo-controlled trial including 24 subjects. Prednisone or placebo was given at a dose of 40 mg per day (8 times higher than in our study) for 8 weeks, followed by a 4-week period of 20 mg per day. The authors observed an increase in CD4 counts (> 40%, p = 0.08) and no changes in (mostly antiretroviral-suppressed) HIV viral load. Two individuals treated with prednisone were found to have asymptomatic osteonecrosis of the hip [33].

Although the CD4-increasing effects of prednisolone in our study were significant, the CD4 gains are low compared to what is achievable with antiretroviral therapy, even in resource-limited settings [34]. Moreover, HIV viral load has approximately tripled in prednisolone-treated study participants and the study failed to show a clinical benefit for the patients in its primary endpoint. And finally, not all potential toxicities of long-term treatment with low-dose prednisolone, including osteoporosis, have been analyzed in this trial. The here-presented data therefore does not justify prednisolone therapy in HIV infection as an alternative to HAART, given both the greater efficacy of HAART, its well-known long-term safety, and its beneficial effects in reducing the risk of HIV transmission [35, 36]. In line with this, the results of this study should not be misinterpreted to diminish our efforts to provide HAART worldwide for everyone.

Chronic immune activation has long been suspected to be a factor in HIV disease progression [2, 3, 5–8, 10, 12, 27, 37, 38]. Although there is an excellent correlation between immune activation and HIV disease progression, there is no direct and definitive proof yet that inhibition of immune activation indeed improves health. The here-presented clinical-endpoint study, in which we investigated the effects of an intervention that directly targets immune activation in HIV infection, provides first data to answer the question of whether a therapeutic decrease of HIV immune activation translates into clinical benefit and may be informative for future clinical trails aiming to develop immune modulatory therapies to be combined with classical HAART.

## Supporting Information

S1 CONSORT Checklist (DOC)

S1 Dataset Minimal Data Set. (XLSX)

S1 File Study Protocol. (PDF)

## References

[1] Serrano-Villar S, Sainz T, Lee SA, Hunt PW, Sinclair E, Shacklett BL, et al HIV-infected individuals with low CD4/CD8 ratio despite effective antiretroviral therapy exhibit altered T cell subsets, heightened CD8+ T cell activation, and increased risk of non-AIDS morbidity and mortality. PLoS pathogens. 2014;10(5):e1004078 10.1371/journal.ppat.1004078 24831517 PMC4022662

[2] Deeks SG, Kitchen CM, Liu L, Guo H, Gascon R, Narvaez AB, et al Immune activation set point during early HIV infection predicts subsequent CD4+ T-cell changes independent of viral load. Blood. 2004;104(4):942–7. Epub 2004/05/01. 10.1182/blood-2003-09-3333 . 15117761

[3] Giorgi JV, Hultin LE, McKeating JA, Johnson TD, Owens B, Jacobson LP, et al Shorter survival in advanced human immunodeficiency virus type 1 infection is more closely associated with T lymphocyte activation than with plasma virus burden or virus chemokine coreceptor usage. J Infect Dis. 1999;179(4):859–70. Epub 1999/03/09. 10.1086/314660 . 10068581

[4] Klatt NR, Funderburg NT, Brenchley JM. Microbial translocation, immune activation, and HIV disease. Trends Microbiol. 2013;21(1):6–13. Epub 2012/10/16. 10.1016/j.tim.2012.09.001 23062765 PMC3534808

[5] Sandler NG, Wand H, Roque A, Law M, Nason MC, Nixon DE, et al Plasma levels of soluble CD14 independently predict mortality in HIV infection. J Infect Dis. 2011;203(6):780–90. Epub 2011/01/22. jiq118 [pii] 10.1093/infdis/jiq118 21252259 PMC3071127

[6] Choudhary SK, Vrisekoop N, Jansen CA, Otto SA, Schuitemaker H, Miedema F, et al Low immune activation despite high levels of pathogenic human immunodeficiency virus type 1 results in long-term asymptomatic disease. J Virol. 2007;81(16):8838–42. Epub 2007/06/01. 10.1128/jvi.02663-06 17537849 PMC1951355

[7] Hunt PW, Brenchley J, Sinclair E, McCune JM, Roland M, Page-Shafer K, et al Relationship between T cell activation and CD4+ T cell count in HIV-seropositive individuals with undetectable plasma HIV RNA levels in the absence of therapy. J Infect Dis. 2008;197(1):126–33. Epub 2008/01/04. 10.1086/524143 18171295 PMC3466592

[8] Hunt PW, Cao HL, Muzoora C, Ssewanyana I, Bennett J, Emenyonu N, et al Impact of CD8+ T-cell activation on CD4+ T-cell recovery and mortality in HIV-infected Ugandans initiating antiretroviral therapy. AIDS. 2011;25(17):2123–31. Epub 2011/09/02. 10.1097/QAD.0b013e32834c4ac1 21881481 PMC3480326

[9] Lyons JL, Uno H, Ancuta P, Kamat A, Moore DJ, Singer EJ, et al Plasma sCD14 is a biomarker associated with impaired neurocognitive test performance in attention and learning domains in HIV infection. J Acquir Immune Defic Syndr. 2011;57(5):371–9. Epub 2011/06/08. 10.1097/QAI.0b013e3182237e54 21646912 PMC3159710

[10] Jiang W, Lederman MM, Hunt P, Sieg SF, Haley K, Rodriguez B, et al Plasma levels of bacterial DNA correlate with immune activation and the magnitude of immune restoration in persons with antiretroviral-treated HIV infection. J Infect Dis. 2009;199(8):1177–85. 10.1086/597476 19265479 PMC2728622

[11] Aiuti F, Mezzaroma I. Failure to reconstitute CD4+ T-cells despite suppression of HIV replication under HAART. AIDS Rev. 2006;8(2):88–97. Epub 2006/07/20. . 16848276

[12] Chahroudi A, Bosinger SE, Vanderford TH, Paiardini M, Silvestri G. Natural SIV hosts: showing AIDS the door. Science. 2012;335(6073):1188–93. Epub 2012/03/10. 10.1126/science.1217550 22403383 PMC3822437

[13] French MA, Cozzi-Lepri A, Arduino RC, Johnson M, Achhra AC, Landay A, et al Plasma levels of cytokines and chemokines and the risk of mortality in HIV-infected individuals: a case-control analysis nested in a large clinical trial. Aids. 2015 10.1097/QAD.0000000000000618 . 25695873 PMC4439268

[14] Hunt PW, Sinclair E, Rodriguez B, Shive C, Clagett B, Funderburg N, et al Gut epithelial barrier dysfunction and innate immune activation predict mortality in treated HIV infection. The Journal of infectious diseases. 2014;210(8):1228–38. 10.1093/infdis/jiu238 24755434 PMC4192038

[15] Tenorio AR, Zheng Y, Bosch RJ, Krishnan S, Rodriguez B, Hunt PW, et al Soluble markers of inflammation and coagulation but not T-cell activation predict non-AIDS-defining morbid events during suppressive antiretroviral treatment. The Journal of infectious diseases. 2014;210(8):1248–59. 10.1093/infdis/jiu254 24795473 PMC4192039

[16] Villar-Garcia J, Hernandez JJ, Guerri-Fernandez R, Gonzalez A, Lerma E, Guelar A, et al Effect of Probiotics (Saccharomyces boulardii) on Microbial Translocation and Inflammation in HIV-Treated Patients: A Double-Blind, Randomized, Placebo-Controlled Trial. Journal of acquired immune deficiency syndromes. 2015;68(3):256–63. 10.1097/QAI.0000000000000468 . 25469528

[17] Kvale D, Ormaasen V, Kran AM, Johansson CC, Aukrust P, Aandahl EM, et al Immune modulatory effects of cyclooxygenase type 2 inhibitors in HIV patients on combination antiretroviral treatment. Aids. 2006;20(6):813–20. 10.1097/01.aids.0000218544.54586.f1 . 16549964

[18] Pettersen FO, Torheim EA, Dahm AE, Aaberge IS, Lind A, Holm M, et al An exploratory trial of cyclooxygenase type 2 inhibitor in HIV-1 infection: downregulated immune activation and improved T cell-dependent vaccine responses. Journal of virology. 2011;85(13):6557–66. 10.1128/JVI.00073-11 21490090 PMC3126508

[19] Overton ET, Sterrett S, Westfall AO, Kahan SM, Burkholder G, Zajac AJ, et al Effects of atorvastatin and pravastatin on immune activation and T-cell function in antiretroviral therapy-suppressed HIV-1-infected patients. Aids. 2014;28(17):2627–31. 10.1097/QAD.0000000000000475 25574964 PMC4338916

[20] Funderburg NT, Jiang Y, Debanne SM, Labbato D, Juchnowski S, Ferrari B, et al Rosuvastatin Reduces Vascular Inflammation and T-cell and Monocyte Activation in HIV-Infected Subjects on Antiretroviral Therapy. Journal of acquired immune deficiency syndromes. 2015;68(4):396–404. 10.1097/QAI.0000000000000478 25514794 PMC4334694

[21] Rajasuriar R, Khoury G, Kamarulzaman A, French MA, Cameron PU, Lewin SR. Persistent immune activation in chronic HIV infection: do any interventions work? AIDS. 2013 Epub 2013/01/18. 10.1097/QAD.0b013e32835ecb8b . 23324661 PMC4285780

[22] Andrieu JM, Lu W. Long-term clinical, immunologic and virologic impact of glucocorticoids on the chronic phase of HIV infection. BMC Med. 2004;2:17 Epub 2004/05/07. 10.1186/1741-7015-2-17 15128452 PMC411065

[23] Andrieu JM, Lu W, Levy R. Sustained increases in CD4 cell counts in asymptomatic human immunodeficiency virus type 1-seropositive patients treated with prednisolone for 1 year. J Infect Dis. 1995;171(3):523–30. Epub 1995/03/01. . 7876597 10.1093/infdis/171.3.523

[24] Ulmer A, Muller M, Bertisch-Mollenhoff B, Frietsch B. Low-dose prednisolone has a CD4-stabilizing effect in pre-treated HIV-patients during structured therapy interruptions (STI). Eur J Med Res. 2005;10(6):227–32. Epub 2005/07/22. . 16033711

[25] Ulmer A, Muller M, Bertisch-Mollenhoff B, Frietsch B. Low dose prednisolone reduces CD4+ T cell loss in therapy-naive HIV-patients without antiretroviral therapy. Eur J Med Res. 2005;10(3):105–9. Epub 2005/04/27. . 15851376

[26] Amadori A, Zamarchi R, De Silvestro G, Forza G, Cavatton G, Danieli GA, et al Genetic control of the CD4/CD8 T-cell ratio in humans. Nature medicine. 1995;1(12):1279–83. . 7489409 10.1038/nm1295-1279

[27] Sidenius N, Sier CF, Ullum H, Pedersen BK, Lepri AC, Blasi F, et al Serum level of soluble urokinase-type plasminogen activator receptor is a strong and independent predictor of survival in human immunodeficiency virus infection. Blood. 2000;96(13):4091–5. Epub 2000/12/09. . 11110678

[28] Addo MM, Altfeld M. Sex-based differences in HIV type 1 pathogenesis. The Journal of infectious diseases. 2014;209 Suppl 3:S86–92. 10.1093/infdis/jiu175 24966195 PMC4157516

[29] Barnighausen T, Chaiyachati K, Chimbindi N, Peoples A, Haberer J, Newell ML. Interventions to increase antiretroviral adherence in sub-Saharan Africa: a systematic review of evaluation studies. The Lancet Infectious diseases. 2011;11(12):942–51. 10.1016/S1473-3099(11)70181-5 22030332 PMC4250825

[30] Fitch KV, Srinivasa S, Abbara S, Burdo TH, Williams KC, Eneh P, et al Noncalcified coronary atherosclerotic plaque and immune activation in HIV-infected women. The Journal of infectious diseases. 2013;208(11):1737–46. 10.1093/infdis/jit508 24041790 PMC3814845

[31] Magee MH, Blum RA, Lates CD, Jusko WJ. Prednisolone pharmacokinetics and pharmacodynamics in relation to sex and race. Journal of clinical pharmacology. 2001;41(11):1180–94. 11697751 10.1177/00912700122012733 PMC4207281

[32] Elliott AM, Luzze H, Quigley MA, Nakiyingi JS, Kyaligonza S, Namujju PB, et al A randomized, double-blind, placebo-controlled trial of the use of prednisolone as an adjunct to treatment in HIV-1-associated pleural tuberculosis. The Journal of infectious diseases. 2004;190(5):869–78. 10.1086/422257 . 15295690

[33] Wallis RS, Kalayjian R, Jacobson JM, Fox L, Purdue L, Shikuma CM, et al A study of the immunology, virology, and safety of prednisone in HIV-1-infected subjects with CD4 cell counts of 200 to 700 mm(-3). J Acquir Immune Defic Syndr. 2003;32(3):281–6. Epub 2003/03/11. . 12626887 10.1097/00126334-200303010-00006

[34] Barth RE, van der Meer JT, Hoepelman AI, Schrooders PA, van de Vijver DA, Geelen SP, et al Effectiveness of highly active antiretroviral therapy administered by general practitioners in rural South Africa. Eur J Clin Microbiol Infect Dis. 2008;27(10):977–84. Epub 2008/07/17. 10.1007/s10096-008-0534-2 . 18629557

[35] Cohen MS, Chen YQ, McCauley M, Gamble T, Hosseinipour MC, Kumarasamy N, et al Prevention of HIV-1 infection with early antiretroviral therapy. N Engl J Med. 2011;365(6):493–505. Epub 2011/07/20. 10.1056/NEJMoa1105243 21767103 PMC3200068

[36] Williams B, Wood R, Dukay V, Delva W, Ginsburg D, Hargrove J, et al Treatment as prevention: preparing the way. J Int AIDS Soc. 2011;14 Suppl 1:S6 Epub 2011/10/05. 1758-2652-14-S1-S6 [pii] 10.1186/1758-2652-14-S1-S6 21967920 PMC3194151

[37] Deeks SG, Walker BD. The immune response to AIDS virus infection: good, bad, or both? J Clin Invest. 2004;113(6):808–10. 10.1172/JCI21318 15067312 PMC362127

[38] Hunt PW. Role of immune activation in HIV pathogenesis. Curr HIV/AIDS Rep. 2007;4(1):42–7. Epub 2007/03/07. . 17338860 10.1007/s11904-007-0007-8

[39] Berkhout B, Bodem J, Erlwein O, Herchenroder O, Khan AS, Lever AM, et al Obituary: Axel Rethwilm (1959–2014). Retrovirology. 2014;11:85 10.1186/s12977-014-0085-9 25270643 PMC4174657

