# Supplementary material for: Effects of Prednisolone on Disease Progression in Antiretroviral-Untreated HIV Infection: A 2-Year Randomized, Double-Blind Placebo-Controlled Clinical Trial
Source: PLoS One. 2016 Jan 26;11(1):e0146678. doi: 10.1371/journal.pone.0146678 (PMC4727920; doi:10.1371/journal.pone.0146678)
Supplement: S1 File — (PDF) [file pone.0146678.s003.pdf]

Progression of HIV-Disease under Low Dose Corticosteroids

PROCORT I - STUDY

A phase II clinical trial to assess risk and benefit of oral low dose prednisolone for HIV infected people prior to the commencement of antiretroviral treatment

**Principal Investigator**	Dr. Samuel Kalluvya, Bugando Medical Center, Mwanza, Tanzania
**Co-Principal Investigator**	PD Dr. August Stich, MD MSc DTM&H<br>Department of Tropical Medicine<br>Medical Mission Institute, Würzburg, Germany
**Co- Investigator**	Prof. Gilbert W. M. Kongola, BUCHS, Mwanza, Tanzania<br>Dr. Charles Majinge, BMC, Mwanza, Tanzania
**Study Coordinator**	Christa Kasang, Clinical Research Assistant, Würzburg,<br>Email: comka@web.de
**Address of Correspondence**:	PD Dr. August Stich, Medical Mission Institute<br>Salvatorstraße 7, 97067 Würzburg<br>Phone: +49 (0)931 / 791 2821<br>Fax: +49 (0)931 / 791 2826<br>Email: stich@missioklinik.de

**Date of version:**	**05.06.2007**

**Scientific Advisory Board:**	Prof. Dr. Hartwig Klinker, Würzburg, Germany<br>Prof. Dr. Klaus Fleischer, Würzburg, Germany<br>Dr. Albrecht Ulmer, Stuttgart, Germany
**Data Safety Monitoring Board :**	Professor Zablon E. Masesa, BUCHS, Mwanza, Tanzania<br>Dr. Koy Mheta, BMC, Mwanza, Tanzania<br>Mr. John Pemba , BMC, Mwanza, Tanzania

TABLE OF CONTENTS

SYNOPSIS **4**

STUDY SITE AND PARTICIPANTS **6**

LIST OF ABBREVIATIONS AND DEFINITIONS OF TERMS **6**

1. INTRODUCTION AND BACKGROUND **7**

1.1 BACKGROUND 7

1.2 RATIONAL FOR THE STUDY 7

1.3. SITE DESCRIPTION 7

2. STUDY OBJECTIVES **8**

2.1 PRIMARY 8

2.2 SECONDARY 8

3. PATIENT DEFINITION **8**

3.1 INCLUSION CRITERIA 8

3.2 EXCLUSION CRITERIA 8

4. PLAN OF THE STUDY **9**

4.1 STUDY DESIGN 9

4.2 STUDY TREATMENT 9

4.3 DOSE MODIFICATION AND DELAY 9

4.4 DISCONTINUATION 9

4.5 PRESTUDY SCREEN 10

4.6 RANDOMIZATION 10

4.7 EVALUATION DURING THE STUDY 10

4.7.1 EVALUATION EVERY VISIT 10

4.7.2 EVALUATION EVERY 3 MONTH 10

4.7.3 EVALUATION EVERY 6 MONTH 10

5. STUDY ASSESSMENTS **11**

5.1 EFFICACY ASSESSMENT 11

5.1.1 TIME TO PROGRESSION 11

5.1.2 AMOUNT OF CD 4-CELLS 11

5.1.3 QUALITY OF LIFE 11

5.1.4 OVERALL SURVIVAL 11

5.2 SAFETY ASSESSMENT METHODS 11
5.2.1 SUSPECTED ADVERSE RESPONSE (SUSAR), ADVERSE EVENT AND TOXICITY 11
5.2.2 LABORATORY MEASUREMENTS 12

6. DATA ANALYSIS / STATISTICAL CONSIDERATIONS 12

7. SERIOUS ADVERSE EVENT MONITORING 13
7.1 DEFINITION OF A SERIOUS ADVERSE EVENT 13
7.2 CAUSALITY ASSESSMENT 13
7.3 REPORTING OF A SERIOUS ADVERSE EVENT 13
7.4 CLINICAL MANAGEMENT OF SERIUOS ADVERSE EVENTS 14

8. STUDY MEDICATION 14
8.1 PREDNISOLONE 14
8.2 PLACEBO 14
8.3 STORAGE 14
8.4 DRUG ACCOUNTABILITY 14

9. INVESTIGATOR OBLIGATIONS 14
9.1 MONITORING, AUDITING AND INSPECTIONS 15
9.2 PATIENT IDENTIFICATION 15
9.3 RECORDING OF DATA 15
9.4 RECORD RETENTION 15
9.5 CONFIDENTIAL FOLLOW-UP 16
9.6 PATIENT INFORMED CONSENT 16
9.7 ETHICAL COMMITTEE/INSTITUTIONAL REVIEW BOARD 16
9.8 DECLARATION OF HELSINKI 16
9.10 INSURANCE OF LIABILITIES 16
9.11 MODIFICATION OF THE PROTOCOL 16
9.12 USE OF INFORMATION AND PUBLICATION 17
9.13 ROLE OF ADVISING BOARD 17
9.14 INVESTIGATOR AGREEMENT 17

10. REFERENCES 18

APPENDICES

APPENDIX Ia: Flow chart

APPENDIX Ib: Study flow sheet

APPENDIX II: WHO Performance Status

APPENDIX III: NCI Common Toxicity criteria

APPENDIX IV: WHO Staging System for HIV Infection and Disease in Adults and Adolescents

APPENDIX Va: CDC Classification for HIV Staging

APPENDIX Vb: WHO Recommendations for initiating antiretroviral therapy in adults and adolescents with documented HIV infection

APPENDIX VI: Informed Consent Form (not yet done)

APPENDIX VII: Serious Adverse Event form (not yet done)

APPENDIX VIII: The Declaration of Helsinki

APPENDIX IX: WHO Guideline of Confirmation for HIV Tests

SYNOPSIS

**Study Title**	A phase II clinical trial to assess risk and benefit of oral low dose prednisolone for HIV infected patients prior to the commencement of antiretroviral treatment
**Principal Investigators**	Dr. Kalluvya, Bugando Medical Centre, Mwanza, Tanzania<br>PD Dr. August Stich<br>Medical Mission Institute Würzburg, Germany
**Co-Investigators**	Prof. Dr. Kongola, BUCHS, Mwanza, Tanzania<br>Dr. Majinge, Bugando Medical Centre, Mwanza, Tanzania
**Study Centre**	Bugando Medical Centre, Mwanza, Tanzania
**Study Period**	QII 2007 – QII 2010
**Objectives**	Primary objective: to assess the effect of the low dose prednisolone on the time to progression of HIV disease;<br><br>Secondary objectives: to evaluate in the same patient population<br>1) amount of CD4-cells<br>2) overall survival<br>3) safety profile<br>4) quality of life
**Methodology**	Mono-centred, double blinded, randomized, phase II placebo-controlled clinical trial of oral low dose prednisolone administration for HIV infected patients in clinical stage CDC A1,2 or B1,2 (see Appendix Va).
**Number of patients**	Maximum of 400 eligible patients in Tanzania, divided in:<br>• 200 patients in study medication group<br>• 200 patients in placebo group
**Inclusion Criteria**	- HIV positivity. The HIV infection has to be confirmed according to WHO guidelines (see Appendix IX).<br>- Patients must have signed a consent form prior to beginning protocol specific procedures (Appendix VI).<br>- Adult male and female patients, age ≥18 years. Female patients of childbearing potential must have a negative pregnancy test at study entry.<br>- Patients must have a stage of HIV disease not yet requiring ARV therapy, defined by CDC stage A1, 2 or B1, 2 (see Appendix Va).<br>- Patients must have a CD4 cell count ≥ 300 cells / µl.<br>- No AIDS defining symptoms.<br>- Patients must have a WHO performance status of 0,1,2 (see Appendix II).

**Exclusion Criteria**	- Prior ARV therapy.<br>- Active tuberculosis.<br>- Abnormal laboratory results especially glucose level >160 mg/dl, liver enzymes AST and/or ALT ≥ 1,5 x ULN, bilirubin ≥ 4 x ULN, alkaline phosphatase ≥ 5 x ULN, creatinine ≥ 2 mg/dl (176,8 µmol)<br>- Serious other diseases including psychiatric disorders.
**Treatment Administration**	Patients in the study group will get prednisolone 5 mg per os daily, patients in the placebo group will get 1 tablet of placebo per os daily.
**Primary Evaluation Criteria**	Effect of the low dose prednisolone therapy on the time to progression. The time to progression is defined as the time between the baseline and the change to CDC stage A3, B3 or C, which indicates the initiation of antiretroviral therapy or the time to death (see Appendix Va).
**Secondary Evaluation Criteria**	• The amount of the CD4 cells at baseline (= 100%) will be compared to the amount during the study.<br>• The overall survival rate will be the time interval between the first dose of the study medication and the date of death. The overall survival will be evaluated in the prolonged follow up period of the study.<br>• The safety profile will be evaluated by clinical examination, adverse events (toxicity), vital signs, haematological and biochemical monitoring.<br>• The quality of life will be evaluated by a Quality of Life Report Form.
**Statistical Methods**	• NQuery 3.0 Test for Patient amount calculation

STUDY SITE AND PARTICIPANTS

Bugando Medical Centre
P.O.Box 1370
Mwanza, Tanzania

Paticipants see front page

LIST OF ABBREVIATIONS AND DEFINITIONS OF TERMS

AE	Adverse Event
ARV	Antiretroviral Drugs
CDC	Centres for Disease Control and Prevention, Atlanta, USA
CRF	Case report form
HAART	Highly active antiretroviral therapy
SAE	Serious adverse event
ULN	Upper laboratory normal
WHO	World Health Organisation

1. INTRODUCTION

1. 1 BACKGROUND

For treatment in advanced HIV infection, combination therapy with antiretroviral drugs (ARVs) is the best option under current international standards. The benefit of ARVs is best, if they are used relatively late in the course of the disease, commonly after a progression of years. However, under a global perspective, more than 90% of those who need ARVs world-wide have no access to them.

ARVs are not used in early stages of infection without significant immunodeficiency. Although a variable degree of immunodeficiency may be present even in early HIV disease, there is no established treatment for this phase.

Immunodeficiency is associated with a decline of a certain population of lymphocytes, the CD4 cells or T-helper cells. It seems to be a useful concept to postulate that a treatment which slows down this loss of CD4 cells could also slow down the progression of HIV disease.

In vitro-findings and clinical studies showed a CD4 cell stabilizing effect of corticosteroids. First clinical studies were based on relatively high dosages (Andrieu JM 1995, LU W 1995, Andrieu JM 2004,). These are hardly justifiable for a longer time, because of numerous side effects, e.g. immunosuppression of the cellular immune system, the manifestation of diabetes or the Cushing syndrome.

In 2005 Ulmer et al. reported in a mono-centric open label study a CD4- stabilizing effect under the administration of low dose prednisolone (5 mg/d) (Ulmer A 2005). Patients with more than 300 CD4 cells/µl were included. After two years the 28 patients on treatment presented 176.1 more CD4 cells than those of 62 control patients in comparison to their baseline counts (p 0.0009) and after three years the 15 patients on treatment presented 236.2 more CD4 cells/µl than those of 33 control patients in comparison to their baseline counts (p = 0,0021). The initiation of treatment with the more expensive and toxic ARVs was delayed.

Based on this study and an increasing amount of experience by Ulmer and others, it seems to be justified to systematically investigate the effect of low dose prednisolone on the progression of HIV disease. The costs of 5 mg prednisolone per day is in a range of 3 US $ per year (Action Medeor). If it turned out that low dose prednisolone prolongs the period until ARVs become indicated without imposing intolerable risks on the patient, this approach could be a very attractive future option for countries with health systems in resource-poor settings.

1.2 RATIONAL FOR THE STUDY

Until now, there are no prospective randomised studies on the use of corticosteroids in latent HIV disease. Furthermore, low dose prednisolone (5 mg/d) is not sufficient tested for the risks and benefit for HIV patients especially for those living in poor settings with a higher risk of infections. This study will assess the benefit and the safety profile for low dose prednisolone therapy for patients in a region with limited resources and high prevalence of infections.

1.3 SITE DESCRIPTION

The study will be conducted at the Bugando Medical Centre in Mwanza, Tanzania.

2. STUDY OBJECTIVES

2.1 PRIMARY

The primary objective of the study is to assess the effect of the low dose prednisolone therapy on the time to progression of HIV disease. The time to progression is defined as the time between the baseline and the change of staging to advanced disease (CDC stage A3, B3 or C) or death (see Appendix Va).

2.2 SECONDARY

The following secondary objectives will be evaluated in the same patient population:

- Amount of CD4 Cells
- Overall survival
- Safety profile
- Quality of life

3. PATIENT DEFINITION

This is a mono-centred trial involving 400 eligible patients.

3.1 INCLUSION CRITERIA

- Positive HIV antibody test. The HIV infection has to be confirmed according to the WHO Guidelines (see Appendix IX).
- Adult male or female patients, age ≥ 18 years.
- Patients must have given an informed consent and signed a consent form prior to beginning protocol specific procedures (Appendix VI).
- Patients in CDC Stage A 1, 2, B 1, 2 (see Appendix Va) not yet requiring ARVs.
- CD4 cell count ≥ 300 cells/µl (for inclusion 300 cells/µl for reason of security).
- No AIDS defining symptoms,
- WHO Performance Status of 0,1,2 (see Appendix II).

3.2 EXCLUSION CRITERIA

- No pregnancy. Female patients of childbearing potential must have a negative pregnancy test at study entry.
- Prior therapy with ARVs.
- Active tuberculosis.
- Abnormal laboratory results especially glucose level > 160 mg/dl, liver enzymes (AST, ALT) ≥ 1,5 x ULN, bilirubin ≥ 4 x ULN, alkaline phosphatase ≥ 5 x ULN, creatinine > 2,0mg/dL (>176,8 µmol).
- Serious other diseases, including psychiatric disorders.

4. PLAN OF STUDY

4.1 STUDY DESIGN

This is a double blinded, randomized, phase II placebo-controlled clinical trial of oral low dose prednisolone therapy for HIV infected patients with CDC Stage A1,2 or B1,2 (see Appendix Va).

The trial is a mono-centred and involves 400 eligible patients in Tanzania, divided in:

- 200 Patients in Study medication group
- 200 Patients in Placebo group

The study enrolment period will last for 12 month (30 patients per month). A study period of 24 month will follow. After 12 months, the first intermediate analysis will be done. In case of no significant negative safety aspects, the study will continue for the next 12 month. A follow-up period for further evaluation can be added.

4.2 STUDY TREATMENT

The patients in the Treatment Group will get prednisolone 5 mg per os daily. The placebo group will get one tablet of placebo per os daily.

4.3 DOSE MODIFICATION AND DELAY

There is no dose modification planned in this study. Study drug related reactions like hypersensitivity reactions, fluid retention, hypertension, gastric ulcer, and diabetes will be treated according to local standards.

4.4 DISCONTINUATION

Patients will be removed from the study for the following reasons:

- disease progression: upgrading of clinical stage and/or CD4 cell count < 200/µl, both requiring the start of HAART (= primary study objective, study completed);
- development of unacceptable toxicity not manageable by symptomatic care;
- administration of antiretroviral treatment;
- significant protocol violation in the prednisolone group, e.g. interruption of taking the study medication for more than four weeks (compliance);
- pregnancy;
- consent withdrawn;
- investigator's decision.

Patients are always allowed to refuse the participation of study and will be withdrawn.

The reason for removal for all patients will be documented on the case report form. Patients who are not eligible or not valuables for the study analysis will be replaced.

4.5 PRE-STUDY SCREEN

The following data will be obtained within two weeks prior to randomization:

- informed consent and signed consent form (audio consent for illiterate persons);
- complete medical history including dates and description of initial diagnosis of HIV infection, concurrent illnesses and concomitant medication;
- physical examination including weight, height, WHO performance status (Appendix II);
- vital signs: blood pressure, pulse rate, oral temperature;
- HIV staging according to CDC: examination for AIDS defining signs and CD4 cell count;
- chest x-ray to exclude active tuberculosis;
- short quality of life score;
- clinical laboratory tests: full blood count, glucose, liver enzymes (AST and/or ALT), bilirubin, alkaline phosphatase , creatinine; pregnancy test for female patients.

4.6 RANDOMIZATION

After having checked the patient's eligibility, the investigator will request randomization. A Randomization list will be provided to the BMC. The randomization number will be given by the study nurse. The study medication is double blind. Block randomization may be used.

4.7 EVALUATION DURING THE STUDY

4.7.1 EVALUATION EVERY VISIT (MONTH 0,1,2,3,4,5,6,9,12,15,18,21,24)

The patient will be examined every month for the following parameters:

- medical history update, including documentation of concurrent conditions, hospitalization and concomitant medications;
- quality of life score;
- physical examination including weight and WHO performance status;
- vital signs: blood pressure, pulse rate and oral temperature;
- HIV staging according to CDC: examination for AIDS defining signs and CD4 cell count;
- full haematological blood count;
- serum chemistry: glucose level, liver enzymes (AST and/or ALT), bilirubin, alkaline phosphatase , creatinine;
- pregnancy test for female patients.
- toxicity evaluation: Toxicity is defined as a study drug related adverse event (AE) grade 3 (severe) or 4 (life threatening). Toxicity will be recorded as it occurs and graded according to the NCI Common Toxicity Criteria (see Appendix III). Toxicities that can not be graded using the NCI Common Toxicity Criteria will be recorded as mild (asymptomatic), moderate (symptomatic but not interfering significantly with function), severe (causing significant interference with function) or life-threatening.

4.7.2 EVALUATION EVERY 3 MONTH (IN ADDITION)

- Sputum test for Tuberculosis.

4.7.3 EVALUATION EVERY 6 MONTH (IN ADDITION)

- Chest x-ray for Tuberculosis.
- Sputum test for Tuberculosis.

5. STUDY ASSESSMENTS

5.1 EFFICACY ASSESSMENT

5.1.1 TIME TO PROGRESSION

The time to progression is defined as time period between the baseline and the change of staging(see appendix Va).

Progression is:

- CD4 cell count ≤ 200 cells/µl; (Stage A3, B3)
- CDC clinical stage C; all counts;
- death.

5.1.2 AMOUNT OF CD4 CELLS

The number of the CD4 cells at baseline (= 100%) will be compared to the number during the study (> 100%: increase; < 100%: decrease).

5.1.3 QUALITY OF LIFE

Some Quality of life questions will be completed for each patient during the visits.

5.1.4 OVERALL SURVIVAL

The duration of survival will be determined by measuring the time interval between the initial dose of study medication and the date of death. The overall survival will be evaluated in the prolonged follow up period of the study.

5.2 SAFETY ASSESSMENT METHODS

5.2.1 SUSPECTED ADVERSE RESPONSE (SUSAR), ADVERSE EVENT AND TOXICITY

Toxicity is defined as a study drug related Suspected Adverse Response (SUSAR) and Adverse events (AE) Grade 3 (severe) or 4 (life threatening). It will be evaluated based on a graded scale of 0-4 using the NCI Common Toxicity Criteria (see Appendix III). Toxicities that can not be graded using the NCI Common Toxicity Criteria will be graded as mild (asymptomatic), moderate (symptomatic but not interfering significantly with function), severe (causing significant interference with function) or life-threatening.

The SUSAR´s are:

Abdominal pain	Hypertension
Diabetes	Hyperglycaemia
Fluid retention	Pneumonia
Gastritis	Weight gain

The AEs are:

Abdominal Pain	Nausea
Candidiasis	Peptic ulcer
Diarrhoea	Pneumonia
Fever	Skin
Herpes simplex,zoster	Vomiting
Hepatitis (liver function)	Urinary tract infection

AEs and SUSARs will be monitored during every visit using a hard coded page in the case report form.

Additional AEs occurring can be added.

5.2.2 LABORATORY MEASUREMENTS

Biochemical and haematological tests will be conducted on blood samples once a month at every visit. If AEs occur additional tests have to be done according to local standards.

6. DATA ANALYSIS / STATISTICAL CONSIDERATIONS

Recruitment time	12 Month
Follow up period	24 Month
Drop-out Rate	10 %
Alpha	0.10
Beta	0.20
Two side test	
Rate of survive (without Progression)	0.791
Rate of survive (without Progression)	0.625

Calculation with NQuery 3.0 Test the result for N is 199 each group.

7. SERIOUS ADVERSE EVENT MONITORING

7.1 DEFINITION OF A SERIOUS ADVERSE EVENT

A serious AE is any adverse drug event occurring that results in any of the following outcomes:
- death
- life-threatening adverse drug experience or event
- in-patient hospitalization or prolongation of existing hospitalization
- a persistent or significant disability/ incapacity
- or a congenital anomaly/birth defect.

- “Life-threatening“ means that the patient was at immediate risk of death from the event as it occurred. It does not include an event that, had it occurred in a more serious form, might have caused death.
- “Requires inpatient hospitalization“ should be defined as hospital admission required for treatment of the adverse event. Hospital admission for scheduled elective surgery would not be a serious adverse event.
- Toxic deaths: Any death occurring during the active treatment part of the study and within 4 weeks following the last study drug intake must be reported to the principal investigator. regardless of any relationship to the study drug(s). Deaths occurring during the study follow-up period of 6 months, need only be reported as adverse events if it’s thought that there is a possible relationship to the study drug(s). Death report form in the CRF must be completed in any case.

7.2 CAUSALITY ASSESSMENT

The assessments to be used to record the causal association between the study drug and the adverse event are as follows:
- no: the adverse event is definitely unrelated to the test drug
- unlikely: the test drug does not have any reasonable association with the observed experienced; however, relationship cannot be definitely excluded
- possible: the connection with test drug administration appears feasible but cannot be concluded with certainty
- probable: the clinical experience appears related to the test drug with a high degree of certainty

For the purposes of regulatory reporting, events which are assessed by the investigator as possibly or probably related to the study drug will normally be considered as indicating a possible association.

7.3 REPORTING OF A SERIOUS ADVERSE EVENT

Any serious event occurring during the active treatment part of the study and within 30 days following the last study drug intake must be reported to the principal investigator within 24 hours, regardless of the relation to study drug(s). Serious adverse events (SAE) probably related to study drugs have to be reported with the yellow form. Study drug related SAE occurring 6 month after the stop of the study need also to be reported and managed without charge.

The study drug related SAE have to be reported to the data safety board every month.

The investigator and others responsible for patient care should institute further supplementary investigations. This may include seeking a further opinion from a specialist in the field of the adverse event. If a patient dies, any post-mortem findings must be recorded.

Withdrawal from the study and therapeutic measures shall be at the discretion of the investigator. A full explanation for the discontinuation from the study will be made at the appropriate case report form. Reporting to local regulatory authorities will take place according to local regulations.

All adverse events, regardless of severity, will be followed up by the investigator until satisfactory resolution.

All other minor adverse reactions will be recorded on the CRF during the study.

7.4 CLINICAL MANAGEMENT OF SERIOUS ADVERSE EVENTS

Study drug related serious adverse events occurring during the study have to be treated at low costs charge free for the patients.

8. STUDY MEDICATION

8.1 PREDNISOLONE

Prednisolone will be dispensed as a varnished tablet of 5 mg produced by Action Medeor, Germany.

8.2 PLACEBO

The placebo will be designed to the equal look like the study medication.

8.3 STORAGE

The storage will take place at room temperature in a dry compartment.

8.4 DRUG ACCOUNTABILITY

The person responsible for drug dispensing is required to maintain adequate records of all study drugs. The patients have to bring back the study medication every month for pill counting. The overall compliance has to be more than 80%.

9. INVESTIGATOR OBLIGATIONS

This study is to be conducted according to globally accepted standards of Good Clinical Practice (ICH, GCP guidelines, current version), in agreement with the latest revision of the Declaration of Helsinki and local regulations.

9.1 MONITORING, AUDITING AND INSPECTIONS

The study will be monitored externally by a study team, who is placed at the Bugando Medical College of Health Sciences in Mwanza, Tanzania, and Medical Mission Hospital in Würzburg, Germany.

During site visits, the monitoring team should review original patient records, drug accountability records and document retention (study file). Additionally, the monitors should observe study procedures and will discuss any problems with the investigator.

Adequate time for these visits should be allocated by the PI. The PI should also ensure that the monitors are given direct access (as per ICH GCP Guideline, Sections 4.9.7 and 6.10) to source documents (i.e. hospital or private charts, original laboratory and for ECG records, appointment books etc.) of the subject which support data entered in the case report forms, as defined in the ICH GCP Guideline, section 1.51 and 1.52.

Participation in this study also implies acceptance of potential inspection by national or foreign health authorities.

9.2 PATIENT IDENTIFICATION

All patients screened for the study will have their initials (first letter of the surname and first letter of first name) and their date of birth entered chronologically in the patient log at the initial visit. In the event a patient is excluded from study participation, the reason is to be documented in the space provided on the patient log.

Each patient will be assigned a Patient Allocation Number on registration. This number and the patient initials are to be entered on the Case Report Form.

9.3 RECORDING OF DATA

Case Report Forms will be supplied by the Study Coordinator at the Medical Mission Hospital. These forms must be <u>PRINTED LEGIBLY</u> using black ball pen.

The forms should be verified against all original records (and workbooks, if applicable) by the Clinical Monitor before submission. The bottom copy will be retained in the investigator‘s files. No case report forms are to be mailed without specific authorization. Case Report Forms and all original data should be readily available for review during scheduled monitoring visits.

9.4 RECORD RETENTION

Copies of all pertinent information will be retained by the investigator for a <u>period of at least 15 years from study completion</u>. Additional considerations must be made about complying with applicable local laws, guidelines, etc.

A study document binder will be provided by the sponsor for all required study documents.

9.5 CONFIDENTIAL FOLLOW-UP

The investigator will be responsible for retaining sufficient information about each patient (e.g. name, address, phone number, social security number, and identity in the study) so that regulatory agencies may access this information should the need to do so arise. These records should be retained in a confidential manner for as long as legally mandated according to local requirements.

9.6 PATIENT INFORMED CONSENT (Appendix VI)

Prior to the screening evaluation, the patient will be informed of the nature of the study drug and will be given pertinent information as to the intended purpose, possible benefits, and possible adverse experiences. The procedures and possible hazards to which the patient is exposed will be explained.

An approved informed consent statement will then be read and signed by the patient, and, when required, a witness, and the investigator. The patient will be provided with a copy of the signed informed consent statement. The patient may withdraw from the study at anytime without prejudicing future medical treatment. Verification of a signed informed consent statement will be noted an the patient‘s study case report form.

9.7 ETHICAL COMMITTEE/INSTITUTIONAL REVIEW BOARD

The final approved protocol and the informed consent statement will be reviewed by a properly constituted Ethics Committee/IRB in Bugando Hospital and the governmental Ethic Committee in Tanzania. The Ethics Committee‘s/Board‘s decision concerning the conduct of the study will be made in writing to the investigator.

The investigator will agree to make required progress reports to the Ethics committee/IRB, as well as report any serious adverse events, life-threatening problems or deaths. The Ethics Committee/IRB must be informed by the investigator of the termination of the study.

9.8 DECLARATION OF HELSINKI

This study is to be performed in accordance with the declaration of Helsinki (Somerset West Amendment, Republic of South Africa Amendment), as described in Appendix VIII.

9.10 INSURANCE OF LIABILITIES

If required, the investigator may forward the Ethics Committee/IRB a copy of the insurance that the sponsor has to take out covering his and any other participating parties liabilities.

9.11 MODIFICATION OF THE PROTOCOL

Any modifications to the protocol which may impact on the conduct of the study, potential benefit of the study, or may affect patient safety, including changes of study objectives, study design, patient population, sample sizes, study procedures, or significant administrative

aspects will require a formal amendment to the protocol. Such amendment will be agreed upon the investigator, and approved by the Ethics Committee/IRB prior to implementation and notified to the health authorities in accordance with local regulations.

Administrative changes of the protocol are minor corrections and/or clarifications that have no effect on the way the study is to be conducted. These administrative changes will be documented in a memorandum. The Ethics Committee/IRB may be notified of administrative changes at the discretion of the investigator.

9.12 USE OF INFORMATION AND PUBLICATION

The information developed during the conduct of this clinical study is considered confidential. This information may be disclosed as deemed necessary by the advisory board.

Should the investigators desire to publish or present the results of this study, a copy of the manuscript or abstract will be provided to the committee of all investigators. The authorship list will be agreed by all investigators prior to publication.

9.13 ROLE OF ADVISORY BOARD

The meeting of the Data Safety monitoring board (DSMB) will be conducted in regular intervals, preferably every six month. The medical advisors will have access to all study materials every time.

9.14 INVESTIGATOR'S AGREEMENT

I have read the preceding protocol dated 2007 and agree that it contains all necessary details for conducting this study. I will conduct the study as outlined therein and will attempt to complete the enrolments into the study. I will provide copies of the protocol and all drug information and prior clinical experience furnished to me, to all physicians responsible to me who participate in the study. I will discuss this material with them to assure that they are fully informed regarding the drug and the conduct of the study. I agree to keep records and all patient information (case report forms and patients' informed consent statement), drug shipment and return forms, and all other information collected during the study in accordance with legal regulations.

Investigator (PRINT NAME):

Investigator Signature

Date

10. REFERENCES

1. Andrieu JM, Lu W, Levy R (1995) Sustained increases in CD4 cell counts in asymptomatic human immunodeficiency virus type 1-seropositive patients treated with prednisolone for 1 year. J Infect Dis 171: 523-530

2. Andrieu JM, Lu W (2004) Long-term clinical, immunologic and virologic impact of glucocorticoids on the chronic phase of HIV infection. BMC Medicine: 2:17

3. CDC (1992) 1993 Revised Classification System for HIV Infection and Expanded Surveillance Case Definition for AIDS Among Adolescents and Adults. MMWR, December 18,1992 / 41 (RR-17)

4. Elliott,AM (2004) A randomized, double-blind, Placebo-controlled Trial of the use of prednisolone as an adjunct to treatment in HIV-1-associated pleural Tuberculosis. JID 190: 869-878;

5. Mayanja-Kizza,H (2005) Immunoadjuvant Prednisolone Therapy for HIV-Associated Tuberculosis: A Phase 2 Clinical Trial in Uganda. JID 191: 856-865

6. Ulmer A (2004) Niedrig dosiertes Prednisolon und viel weniger Angewiesenheit auf HAART – ein anderer Therapieansatz, in: Jäger, H.: AIDS und HIV-Infektionen: Diagnostik, Klinik, Behandlung; Handbuch und Atlas für Klinik und Praxis. ecomed-Verlag, Landsberg, 43.Erg.Lfg.3/04 VII-10.2, 1-7

7. Ulmer,A (2005) Low dose prednisolone Reduces CD4+ T-Cell loss in Therapy-naïve HIV-Patients without antiretroviral Therapy. Eur. J..Med.Res. 10:105-109

8. Wallis RS, Kalyjian R, Jacobsen JM, Fox L, Purdue L, Shikuma CM, Arakaki R, Snyder S, Coombs RW, Bosch RJ, Spritzler J, Chernoff M, Aga E, Myers L, Schock B, Ledermann MM (2003) A Study of the Immunology, Virology, and Safety of prednisolone in HIV-1-Infected Subjects with CD4 Cell Counts of 200 to 700mm3. JAIDS 32: 281-286

9. Wellcome Trust: Research involving people living in Developing Countries

10. Lu W, Salerno-Goncalves R, Yuan J, Sylvie D, Han DS, Andrieu JM. Glucocorticoids rescue CD4+ T lymphocytes from activation-induced apoptosis triggered by HIV-1: implications for pathogenesis and therapy. AIDS. 1995;**9**:35–42.

11. Wu AW, Hays, RD, Kelly S, Malitz F, Bozzette SA, (1997a) Applications of the Medical Outcomes Study: health-related quality of life measures in HIV/AIDS. Quality of life research, 6, 531-554.

12. Wu AW, Revicki DA, Jacobson D, Malitz F, (1997b) Evidence for reliability, validity and usefulness of the Medical Outcomes Study HIV Health survey (MOS-HIV). Quality of life research, 6, 481-493.

13. Wu AW, Rubin HR, Mathews WC, Ware JE, Brysk LT, Hardy WD, Bozzette SA, Spectors SA, Richman DD, (1991) A health status questionnaire using 30 items from the Medical Outcomes Study. Preliminary validation in persons with early HIV infection. Medical care, 29: 786-798.

Appendix Ia

FLOW CHART

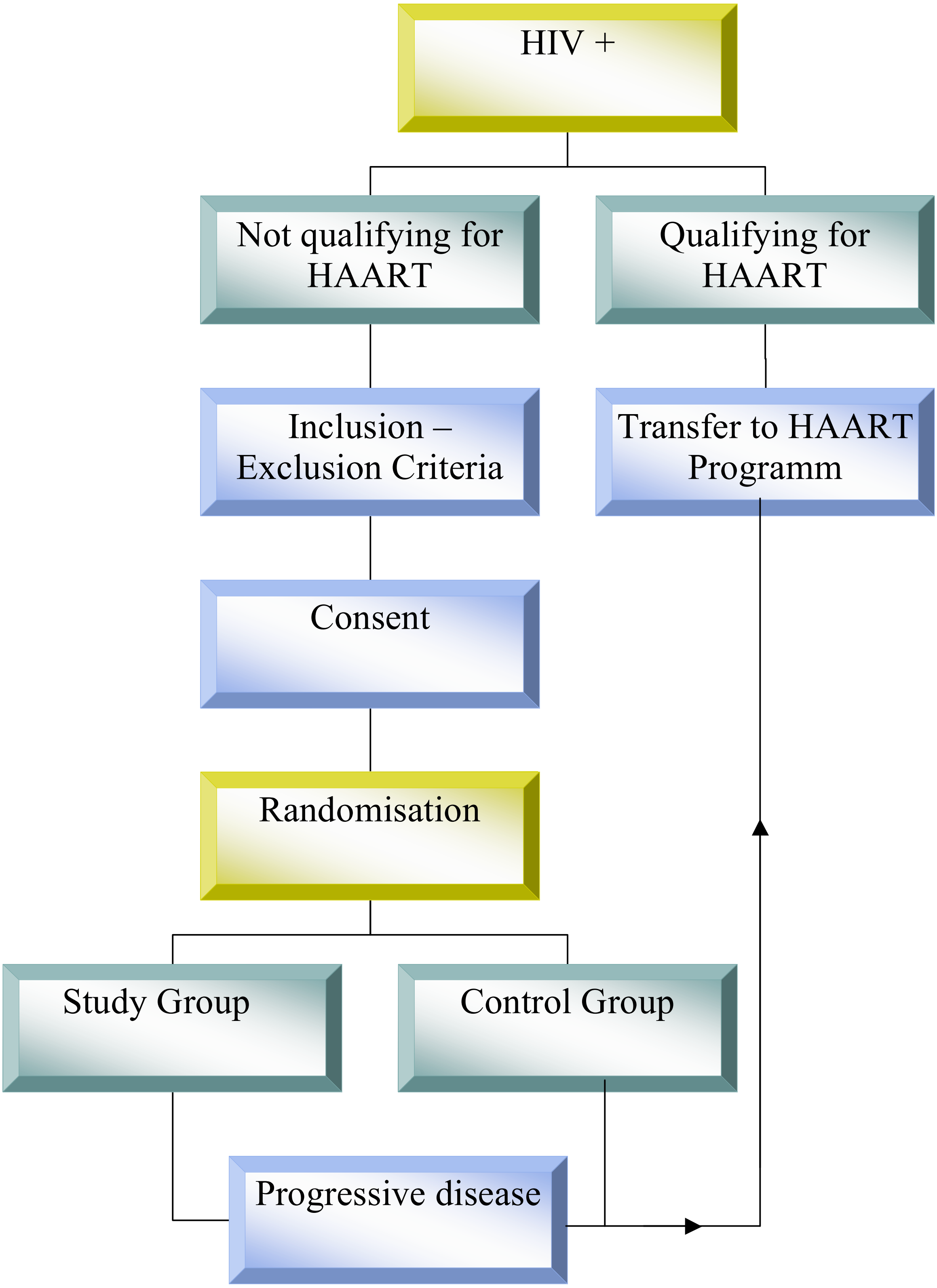

Appendix Ib

STUDY FLOW SHEET

VISIT	Base-line	Study start	1	2	3	4	5	6	7	8	9	10
MONTH	Minus 1	0	1st month	2nd month	3rd month	4th month	5th month	6th month	9th month	12th month	15th month	18th month
Informed Consent	x											
Quality of life	x	x	x	x	x	x	x	x	x	x	x	x
Physical examination	x	x	x	x	x	x	x	x	x	x	x	x
Vital signs	x	x	x	x	x	x	x	x	x	x	x	x
Height, Weight	x	x	x	x	x	x	x	x	x	x	x	x
WHO perf. Status	x	x	x	x	x	x	x	x	x	x	x	x
Staging CDC	x	x	x	x	x	x	x	x	x	x	x	x
Concomitant disease	x											
Concomitant medication	X	x	x	x	x	x	x	x	x	x	x	x
Signs & Symptoms		x	x	x	x	x	x	x	x	x	x	x
Glucose (Nurse)	x	x	x	x	x	x	x	x	x	x	x	x
Pregnancy test (Doc. As)	x	x	x	x	x	x	x	x	x	x	x	x
Chest x-ray (x-ray dep.)	x							x		x		x
TB sputum (TB Lab.)	x				x			x	x	x	x	x
Whole blood count & PLTs	x	x	x	x	x	x	x	x	x	x	x	x
CD4 count	x	x	x	x	x	x	x	x	x	x	x	x
HIV Confirnation	x											
Biochemistry examinations	x	x	x	x	x	x	x	x	x	x	x	x
CPT preparation		x	x		x			x		x		

Appendix II WHO Performance Status

0 Able to carry out all normal activity without restriction.

1 Restricted in physically strenuous activity but ambulatory and able to do light work.

2 Ambulatory and capable of all self-care but unable to carry out any work. Up and about more than 50 % of waking hours.

3 Capable of only limited self-care, confined to bed or chair more than 50 % of waking hours.

4 Completely disabled: Cannot carry on any self-care, totally confined to bed or chair.

Appendix III NCI COMMON TOXICITY CRITERIA

	Grade				
TOXICITY	0	1	2	3	4
WBC (10^9/l)	≥ 4.0	3.0 - 3.9	2.0 - 2.9	1.0 - 1.9	< 1.0
PLT (10^9/l)	WNL	75.0 - normal	50.0 - 74.9	25.0 - 49.9	< 25.0
Hgb (g/dl)	WNL	10.0 - normal	8.0 - 9.9	6.5 - 7.9	< 6.5
Granulocytes/ Bands (10^9/l)	≥ 2.0	1.5 - 1.9	1.0 - 1.4	0.5 - 0.9	< 0.5
Lymphocytes (10^9/l)	≥ 2.0	1.5 - 1.9	1.0 - 1.4	0.5 - 0.9	< 0.5
Hemorrhage (clinical)	None	mild, no transfusion	gross, 1-2 units transfusion per episode	gross, 3-4 units transfusion per episode	massive > 4 units transfusion per episode
Infection	None	mild	moderate	severe	life-threatening
Nausea	None	able to eat reasonable intake	intake significantly decreased but can eat	no significant intake	--
Vomiting	None	1 episode in 24 hrs	2-5 episodes in 24 hrs	6-10 episodes in 24 hrs	> 10 episodes in 24 hrs or requiring parenteral support
Diarrhea	None	increase of 2-3 stools/day over pre-Rx	increase of 4-6 stools/day, or nocturnal stools, or moderate cramping	increase of 7-9 stools/day, or incontinence, or severe cramping	increase of ≥ 10 stools/day, or grossly bloody diarrhea or need parenteral support
Stomatitis	None	painless ulcers, erythema, or mild soreness	painful erythema, edema, or ulcers, but can eat	painful erythema, edema, or ulcers, and cannot eat	requires parenteral or enteral support
Bilirubin	WNL	--	< 1.5 x N	1.5 - 3.0 x N	> 3.0 x N
Transaminase (SGOT, SGPT)	WNL	≤ 2.5 x N	2.6 - 5.0 x N	5.1 - 20.0 x N	> 20.0 x N
Alk. Phos. or 5' nucleotidase	WNL	≤ 2.5 x N	2.6 - 5.0 x N	5.1 - 20.0 x N	> 20.0 x N
Liver - clinical	no change from baseline	--	--	precoma	hepatic coma
Creatinine	WNL	< 1.5 x N	1.5 - 3.0 x N	3.1 - 6.0 x N	> 6.0 x N
Proteinuria	no change	1+ or < 0.3 g% or < 3 g/1	2 - 3+ or 0.3 - 1.0 g% or 3 - 10 g/1	4+ or > 1.0 g% or > 10 g/1	nephrotic syndrome
Hematuria	Neg	micro only	gross, no clots	gross + clots	requires transfusion
Alopecia	no loss	mild hair loss	pronounced or total hair loss	--	--
Pulmonary	none or no change	asymptomatic, w. abnormality in PFT's	dyspnea on significant exertion	dyspnea at normal level of activity	dyspnea at rest

Cardiac dysrhythmias	None	asymptomatic, transient, requiring no therapy	recurrent or persistent, no therapy required	requires treatment	requires monitoring ; or hypotension, or ventricular tachycardia, or fibrillation
Cardiac function	None	asymptomatic decline of resting LVEF less than 20% of baseline value	asymptomatic decline of resting LVEF more than 20% of baseline value	mild CHF, responsive to therapy	severe or refractory CHF
Cardiac-Ischemia	None	non-specific T-wave flattening	asymptomatic, ST and T wave changes suggesting ischemia	angina without evidence for infarction	acute myocardial infarction
Cardiac-Pericardial	None	asymptomatic effusion, no intervention required	pericarditis (rub, chest pain ECG changes)	symptomatic effusion ; drainage required	tamponade ; drainage urgently required
Hypertension	none or no change	asymptomatic, transient increase by > 20 mmHg (D) or to > 150/100 if previously WNL. No treatment required	recurrent or persistent increase by > 20 mmHg (D) or to > 150/100 if previously WNL. No treatment required	requires therapy	hypertensive crisis
Hypotension	none or no change	changes requiring no therapy (incl. transient orthostatic hypotension)	requires fluid replacement or other therapy but not hospitalization	requires therapy and hospitalization resolves within 48 hrs of stopping the agent	requires therapy and hospitalization for > 48 hrs after stopping the agent
Neuro-sensory	none or no change	mild paresthesias loss of deep tendon reflexes	mild or moderate objective sensory loss moderate paresthesias	severe objective sensory loss or paresthesias that interfere with function	--
Neuro-motor	none or no change	subjective weakness: no objective findings	mild objective weakness without significant impairment of function	objective weakness with impairment of function	paralysis
Neuro-cortical	None	mild somnolence or agitation	moderate somnolence or agitation	severe somnolence, agitation, confusion, disorientation or hallucinations	coma, seizures, toxic psychosis
Neuro-cerebellar	None	slight, incoor-dination, dys-diadokinesis	intention tremor, dysmetria, slurred speech, nystagmus	locomotor ataxia	cerebellar necrosis
Neuro-mood	no change	mild anxiety or depression	moderate anxiety or depression	severe anxiety or depression	suicidal ideation
Neuro-headache	None	mild	moderate or severe but transient	unrelenting and severe	--
Neuro-constipation	none or no change	mild	moderate	severe	ileus > 96 hrs
Neuro-hearing	none or no change	asymptomatic, hearing loss on audiometry only	tinnitus	hearing loss interfering with function but correctable with hearing aid	deafness not correctable

Neuro- vision	none or no change	--	--	symptomatic subtotal loss of vision	blindness
Skin	none or no change	scattered macular or papular eruption or erythema that is asymptomatic	scattered macular or papular eruption or erythema with pruritus or other associated symptoms	generalized symptomatic macular, papular, or vesicular eruption	exfoliative dermatitis or ulcerating dermatitis
Allergy	None	transient rash drug fever < 38°C, 100.4°F	urticaria, drug fever = 38°C, 100.4°F mild bronchospasm	serum sickness bronchospasm, req. parenteral meds	anaphylaxis
Fever in absence Of infection	None	37.1 - 38.0°C 98.7 - 100.4°F	38.1 - 40.0°C 100.5 - 104.0°F	> 40.0°C > 104.0°F for less than 24 hrs	> 40.0°C (104.0°F) for more than 24 hrs or fever accompanied by hypotension
Local	None	pain	pain with swelling with inflammation or phlebitis	ulceration	plastic surgery indicated
Weight gain / Loss	< 5.0%	5.0 - 9.9%	10.0 - 19.9%	≥ 20.0%	--
Hyperglycemia (mg/dl)	< 116	116 - 160	161 - 250	251 - 500	> 500 or ketoacidosis
Hypoglycemia (mg/dl)	> 64	55 - 64	40 - 54	30 - 39	< 30
Amylase	WNL	< 1.5 x N	1.5 - 2.0 x N	2.1 - 5.0 x N	≥ 5.1 x N
Hypercalcemia (mg/dl)	< 10.6	10.6 - 11.5	11.6 - 12.5	12.6 - 13.5	≥ 13.5
Hypocalcemia (mg/dl)	> 8.4	8.4 - 7.8	7.7 - 7.0	6.9 - 6.1	≤ 6.0
Hypomagnesemia (meq/l)	> 1.4	1.4 - 1.2	1.1 - 0.9	0.8 - 0.6	≤ 0.5
Fibrinogen	WNL	0.99 - 0.75 x N	0.74 - 0.50 x N	0.49 - 0.25 x N	≤ 0.24 x N
Prothrombin time	WNL	1.01 - 1.25 x N	1.26 - 1.50 x N	1.51 - 2.00 x N	> 2.00 x N
Partial thromboplastin time	WNL	1.01 - 1.66 x N	1.67 - 2.33 x N	2.34 - 3.00 x N	> 3.00 x N

Appendix IV

WHO Recommendations for initiating antiretroviral therapy in adults and adolescents with documented HIV infection

If CD4 testing available:

- WHO Stage IV disease irrespective of CD4 cell count
- WHO Stage III disease (including but not restricted to HIV wasting, chronic diarrhoea of unknown aetiology, prolonged fever of unknown eatiology, active pulmonary tuberculosis, recurrent invasive Bacterial infections, or recurrent/persistent mucosal candidiasis) with consideration of using CD4 cell counts < 350/mm3 to assist decision making
- WHO Stage I or II disease with CD4 cell counts ≤ 200/mm3

Appendix Va

1993 Revised Classification System for HIV Infection and the Expanded CDC Surveillance Case Definition of AIDS in Adults and Adolescents

	Clinical Categories		
**CD4 T-cell Categories**	**A**<br>**A**symptomatic	**B**<br>**S**ymptomatic	**C**<br>AIDS Indicator Conditions
>500/mm3	A1	B1	C1
200-499/mm3	A2	B2	C2
<200/mm3	A3	B3	C3

1993 CDC Surveillance Case Definition		
**Category A**<br>(Asymptomatic HIV)	- Asymptomatic HIV Infection<br>- Persistent generalized lymphadenopathy (PGL)<br>- Acute HIV Infection with accompanying illness or history of HIV infection	
**Category B**<br>(Symptomatic HIV)	- Bacillary angiomatosis<br>- Candidiasis, oropharyngeal (thrush), vulvovaginal (>1month)<br>- Cervical dysplasia<br>- Constitutional symptoms (fever >38 C or diarrhea >1 month)<br>- Hairy leukoplakia	- Herpes zoster (shingles) involving at least 2 distinct episodes or more than 1 dermatome<br>- Idiopathic thrombocytopenia purpura<br>- Listeriosis<br>- Pelvic Inflammatory Disease<br>- Peripheral Neuropathy
**Category C**<br>(AIDS Defining Infections)	- Candidiasis of bronchi, trachea, or lungs<br>- Candidiasis, oesophageal<br>- Cervical cancer, invasive<br>- Coccidioidomycosis, disseminated or extrapulmonary<br>- Cryptococcosis, extrapulmonary<br>- Cryptosporidiosis, chronic (>1 month)<br>- Cytomegalovirus disease or retinitis<br>- Encephalopathy, HIV related<br>- Herpes simplex virus (HSV)<br>- Histoplasmosis, disseminated or extrapulmonary<br>- HIV-associated dementia<br>- Kaposi sarcoma	- Lymphoid interstitial pneumonia<br>- Lymphoma, Burkitt's, immunoblastic, primary of brain<br>- Mycobacterium avium-intracellulare complex<br>- Mycobacterium tuberculosis, pulmonary or extrapulmonary<br>- Nocardiosis<br>- Pneumocystis carinii pneumonia<br>- Pneumonia, recurrent<br>- Progressive multifocal leukoencephalopathy<br>- Salmonella septicemia, recurrent<br>- Toxoplasmosis of internal organs<br>- Wasting syndrome due to HIV<br>- Isosporiasis, chronic intestinal (>1 month)

Appendix Vb

WHO Staging System for HIV Infection and Disease in Adults and Adolescents

Clinical Stage I

1. Asymptomatic
2. Generalised lymphadenopathy

Performance scale: 1: asymptomatic, normal activity

Clinical Stage II

3. Weight loss, < 10% of body weight
4. Minor mucocutaneous manifestations (seborrheic dermatitis, prurigo, fungal nail infections, recurrent oral ulcerations, angular cheilitis)
5. Herpes zoster within the last five years
6. Recurrent upper respiratory tract infections (i.e. bacterial sinusitis)

And/or performance scale 2: symptomatic , normal activity

Clinical Stage III

7. Weight loss, < 10% of body weight
8. Unexpalined chronic diarroea, > 1 month
9. Unexplained prolonged fever (intermitent or constant) , > 1 month
10. Oral candidiasis (thrush)
11. Oral hairy leucoplakia
12. Pulmonary tuberculosis within the past year
13. Severe bacterial infections (i.e. pneumonia, pyomyositis)

And/or performance scale 3: bedridden < 50% of the day during last month.

Clinical Stage IV:

14. HIV wasting syndrome (1)
15. Pneumocystic carinii pneumonia
16. Toxoplasmosis of the brain
17. Cryptosporidiosis with diarrhoea > 1 month
18. Cryptococcosis, extrapulmonary
19. Cytomegalovirus disease of an organ other than liver, spleen or lymph node (ex: retinitis)
20. Herpes simplex virus infection, mucocutaneous (>1month) or visceral
21. Progressive multifocal leucoencephalopathy
22. Any dissiminated endemic mycosis
23. Candidiasis of esophagus, trachea, bronchi
24. Atypical mycobacteriosis, disseminated or lungs
25. Non-typhoid Salmonella septicemia
26. Extrapulmonary tuberculosis
27. Lymphoma
28. Kaposi's sarcoma
29. HIV encephalopathy (2)

And/or performance scale 4: bedridden > 50% of the day during last month.

(1) HIV wasting syndrome: weight loss of > 10% of body weight, plus either unexplained chronic diarrohea (> 1 month) or chronic weakness and unexplained prolonged fever (> 1 month).

(2) HIV encephalopathy: clinical findings of disabling cognitive and/or motor dysfunction interfering with activities of daily living, progressing over weeks to months, in the absence of a concurrent illness or condition other than HIV infection which could explain the findings.

Appendix VI

Informed Consent Form

PATIENT INFORMATION SHEET & CONSENT FORM

Study title

A phase II clinical trial to assess risk and benefit of oral low dose prednisolone for HIV infected people prior to the commencement of antiretroviral treatment.

Invitation paragraph

You are being invited to take part in a research study. Before you decide, it is important for you to understand why the research is being done and what it will involve. Please take time to read the following information carefully and discuss it with others if you wish. Ask us if there is anything that is not clear or if you would like more information. Take time to decide whether or not you wish to take part.

Thank you for reading this.

Why is this study being done?

The purpose of this study is to find out whether the study medication Prednisolone 5 mg is able to stabilize your condition. Prednisolone has been shown to protect the immune system from self-destruction, so we hope that this effect will prolong the time of health in your situation. Prednisolone is a well known medication for treating allergy. In prior studies with HIV-infected people prednisolone has been shown to have a stabilizing effect on health.

In this study we will compare the effects, good and/or bad, of prednisolone 5 mg with placebo.

This study is a double blind study with either placebo medication or with prednisolone 5mg per day. This means that only the registration centre knows if you get study medication (group 1) or placebo (group2). You will be "randomized" into one of the study groups described below. Randomization means that you are put into a group by chance. A computer program will place you in one of the study groups. Neither you nor your doctor can choose the group you will be in. You will have an equal chance of being placed in either group.

Do I have to take part?

It is up to you to decide whether or not to take part. If you do decide to take part you will be given this information sheet to keep and be asked to sign a consent form. If you decide to take part you are still free to withdraw at any time and without giving a reason. A decision to withdraw at any time, or a decision not to take part, will not affect the standard of care you receive.

How many people will take part in the study?

About 400 people will take part in this study.

What will happen if I take part in this research study?

Before you begin the study …

You will need to have the following exams, tests or procedures to find out if you can be in the study. These exams, tests or procedures are part of regular diagnostic procedures and may be done even if you do not join the study. If you have had some of them recently, they may not need to be repeated. This will be up to your study doctor.

- *HIV Test*
- *Blood sample*
- *For female patients, Pregnancy test*
- *Chest x-ray for tuberculosis diagnostic*
- *Sputum test*

During the study …

If the exams, tests and treatment medication show that you can be in the study, and you choose to take part, then you will need the following tests and procedures. They are part of regular care.

- *Monthly blood samples for six months then quarterly*
- *Chest x-ray every half year*
- *Sputum test every three months*
- *Pregnancy test every half year*

Additional Laboratory Analysis: For later additional laboratory analysis we will store a small amount of your blood samples at the National Institute for Medical Research. These blood samples will be analyzed in Germany (Medical Mission Institute) to help the doctors to understand how the drug is working on your cells.

How long will I be in the study?

You will be asked to take prednisolone for two years. After you are finished taking prednisolone, the study doctor will ask you to come for one further visit one month after you stop the study medication.

Can I stop being in the study?

Yes. You can decide to stop at any time. Tell the study doctor if you are thinking about stopping or decide to stop. He or she will tell you how to stop safely.

It is important to tell the study doctor if you are thinking about stopping so any risks from the study medication can be evaluated by your doctor. Another reason to tell your doctor that you are thinking about stopping is to discuss how to go onto another therapy, instead of prednisolone.

The study doctor may stop you from taking part in this study at any time if he/she believes it is in your best interest; if you do not follow the study rules; or if the study is stopped.

What side effects or risks can I expect from being in the study?

Prednisolone given in low dose is known as a medication with few side effects. It has been proven in several other studies to be safe in this dosage. But you may have side effects while on the study. Everyone taking part in the study will be watched carefully for any side effects. However, doctors don't know all the side effects that may happen. Side effects may be mild or very serious. Your health care team may give you medicines to help lessen side effects. Many side effects go away soon after you stop taking the study medication.
You should talk to your study doctor about any side effects that you have while taking part in the study.

Risks and side effects related to the prednisolone include those which are:

**Likely**	**Less Likely**	**Rare but serious**
**- Gastritis**	**- Hypertension**	**- Pneumonia**
**- Abdominal pain**	**- Fluid retention**	**- Diabetes**
**- Weight gain**	**- Hyperglycaemia**	

Side effects reported by researchers but not proven to be caused by prednisolone

- **Trigger the possibility to get a Kaposi sarcoma**

Reproductive risks: You should not become pregnant or father a baby while on this study because the drugs in this study can affect an unborn baby.

For more information about risks and side effects, ask your study doctor.

Are there benefits to taking part in the study?

Taking part in this study may or may not make your health better. While doctors hope prednisolone therapy will be more useful to stabilize your health compared to the usual treatment, there is no proof of this yet. We do know that the information from this study will help doctors learn more about prednisolone as a treatment for HIV infected patients. This information could help future patients.

What other choices do I have if I do not take part in this study?

Your other choices may include:

- **Getting treatment or care for your HIV Infection without being in a study**
- **Getting no treatment.**

Talk to your physician about your hoices and how to obtain care before you decide if you will take part in this study.

Will my medical information and my taking part in this study be kept confidential?

All information which is collected about you during the course of the research will be kept strictly confidential. Any information about you which leaves the hospital/surgery will have your name and address removed so that you cannot be recognized by a third party.

What are the costs of taking part in this study?

You will not need to pay for the study medication and for all laboratory tests and examinations which are study specific procedures.

You will not be paid for taking part in this study but you will get the bus transfer paid.

What happens if I am injured because I took part in this study?

It is important that you tell your study doctor if you feel that you have been injured because of taking part in this study. You can tell the doctor in person or call him/her at the Bugando hospital .

You will get medical treatment if you are injured as a result of taking part in this study.

What are my rights if I take part in this study?

Taking part in this study is your choice. You may choose either to take part or not to take part in the study. If you decide to take part in this study, you may leave the study at any time. No matter what decision you make, there will be no penalty to you and you will not lose any of your regular benefits. Leaving the study will not affect your medical care. You can still get your medical care from our institution.

We will tell you about new information or changes in the study that may affect your health or your willingness to continue in the study.

In the case of injury resulting from this study, you do not lose any of your legal rights to seek payment by signing this form.

Who can answer my questions about the study?

You can talk to your study doctor about any questions or concerns you have about this study. Contact your study doctor at Bugando hospital.

Please give your consent

Signature

I have been given a copy of all 6 pages of this form. I have read it or it has been read to me. I understand the information and have had my questions answered. I agree to take part in this study.

Participant: ________________________________ Study No.__________

Date ____________________________________

Investigator: ________________________________

Date ____________________________________

Appendix VII

Serious Adverse Event Form

The tansanian yellow form

Appendix VIII

The Declaration of Helsinki

Policy

WORLD MEDICAL ASSOCIATION DECLARATION OF HELSINKI
Ethical Principles for Medical Research Involving Human Subjects

Adopted by the 18th WMA General Assembly, Helsinki, Finland, June 1964, and amended by the
29th WMA General Assembly, Tokyo, Japan, October 1975
35th WMA General Assembly, Venice, Italy, October 1983
41st WMA General Assembly, Hong Kong, September 1989
48th WMA General Assembly, Somerset West, Republic of South Africa, October 1996
and the 52nd WMA General Assembly, Edinburgh, Scotland, October 2000
Note of Clarification on Paragraph 29 added by the WMA General Assembly, Washington 2002
Note of Clarification on Paragraph 30 added by the WMA General Assembly, Tokyo 2004

A. INTRODUCTION

1. The World Medical Association has developed the Declaration of Helsinki as a statement of ethical principles to provide guidance to physicians and other participants in medical research involving human subjects. Medical research involving human subjects includes research on identifiable human material or identifiable data.
2. It is the duty of the physician to promote and safeguard the health of the people. The physician's knowledge and conscience are dedicated to the fulfillment of this duty.
3. The Declaration of Geneva of the World Medical Association binds the physician with the words, "The health of my patient will be my first consideration," and the International Code of Medical Ethics declares that, "A physician shall act only in the patient's interest when providing medical care which might have the effect of weakening the physical and mental condition of the patient."
4. Medical progress is based on research which ultimately must rest in part on experimentation involving human subjects.
5. In medical research on human subjects, considerations related to the well-being of the human subject should take precedence over the interests of science and society.
6. The primary purpose of medical research involving human subjects is to improve prophylactic, diagnostic and therapeutic procedures and the understanding of the aetiology and pathogenesis of disease. Even the best proven prophylactic,

diagnostic, and therapeutic methods must continuously be challenged through research for their effectiveness, efficiency, accessibility and quality.
7. In current medical practice and in medical research, most prophylactic, diagnostic and therapeutic procedures involve risks and burdens.
8. Medical research is subject to ethical standards that promote respect for all human beings and protect their health and rights. Some research populations are vulnerable and need special protection. The particular needs of the economically and medically disadvantaged must be recognized. Special attention is also required for those who cannot give or refuse consent for themselves, for those who may be subject to giving consent under duress, for those who will not benefit personally from the research and for those for whom the research is combined with care.
9. Research Investigators should be aware of the ethical, legal and regulatory requirements for research on human subjects in their own countries as well as applicable international requirements. No national ethical, legal or regulatory requirement should be allowed to reduce or eliminate any of the protections for human subjects set forth in this Declaration.

B. BASIC PRINCIPLES FOR ALL MEDICAL RESEARCH

10. It is the duty of the physician in medical research to protect the life, health, privacy, and dignity of the human subject.
11. Medical research involving human subjects must conform to generally accepted scientific principles, be based on a thorough knowledge of the scientific literature, other relevant sources of information, and on adequate laboratory and, where appropriate, animal experimentation.
12. Appropriate caution must be exercised in the conduct of research which may affect the environment, and the welfare of animals used for research must be respected.
13. The design and performance of each experimental procedure involving human subjects should be clearly formulated in an experimental protocol. This protocol should be submitted for consideration, comment, guidance, and where appropriate, approval to a specially appointed ethical review committee, which must be independent of the investigator, the sponsor or any other kind of undue influence. This independent committee should be in conformity with the laws and regulations of the country in which the research experiment is performed. The committee has the right to monitor ongoing trials. The researcher has the obligation to provide monitoring information to the committee, especially any serious adverse events. The researcher should also submit to the committee, for review, information regarding funding, sponsors, institutional affiliations, other potential conflicts of interest and incentives for subjects.
14. The research protocol should always contain a statement of the ethical considerations involved and should indicate that there is compliance with the principles enunciated in this Declaration.
15. Medical research involving human subjects should be conducted only by scientifically qualified persons and under the supervision of a clinically competent medical person. The responsibility for the human subject must always rest with a medically qualified person and never rest on the subject of the research, even though the subject has given consent.
16. Every medical research project involving human subjects should be preceded by careful assessment of predictable risks and burdens in comparison with foreseeable benefits to the subject or to others. This does not preclude the participation of healthy volunteers in medical research. The design of all studies should be publicly available.
17. Physicians should abstain from engaging in research projects involving human subjects unless they are confident that the risks involved have been adequately assessed and can be satisfactorily managed. Physicians should cease any investigation if the risks are found to outweigh the potential benefits or if there is conclusive proof of positive and beneficial results.

18. Medical research involving human subjects should only be conducted if the importance of the objective outweighs the inherent risks and burdens to the subject. This is especially important when the human subjects are healthy volunteers.
19. Medical research is only justified if there is a reasonable likelihood that the populations in which the research is carried out stand to benefit from the results of the research.
20. The subjects must be volunteers and informed participants in the research project.
21. The right of research subjects to safeguard their integrity must always be respected. Every precaution should be taken to respect the privacy of the subject, the confidentiality of the patient's information and to minimize the impact of the study on the subject's physical and mental integrity and on the personality of the subject.
22. In any research on human beings, each potential subject must be adequately informed of the aims, methods, sources of funding, any possible conflicts of interest, institutional affiliations of the researcher, the anticipated benefits and potential risks of the study and the discomfort it may entail. The subject should be informed of the right to abstain from participation in the study or to withdraw consent to participate at any time without reprisal. After ensuring that the subject has understood the information, the physician should then obtain the subject's freely-given informed consent, preferably in writing. If the consent cannot be obtained in writing, the non-written consent must be formally documented and witnessed.
23. When obtaining informed consent for the research project the physician should be particularly cautious if the subject is in a dependent relationship with the physician or may consent under duress. In that case the informed consent should be obtained by a well-informed physician who is not engaged in the investigation and who is completely independent of this relationship.
24. For a research subject who is legally incompetent, physically or mentally incapable of giving consent or is a legally incompetent minor, the investigator must obtain informed consent from the legally authorized representative in accordance with applicable law. These groups should not be included in research unless the research is necessary to promote the health of the population represented and this research cannot instead be performed on legally competent persons.
25. When a subject deemed legally incompetent, such as a minor child, is able to give assent to decisions about participation in research, the investigator must obtain that assent in addition to the consent of the legally authorized representative.
26. Research on individuals from whom it is not possible to obtain consent, including proxy or advance consent, should be done only if the physical/mental condition that prevents obtaining informed consent is a necessary characteristic of the research population. The specific reasons for involving research subjects with a condition that renders them unable to give informed consent should be stated in the experimental protocol for consideration and approval of the review committee. The protocol should state that consent to remain in the research should be obtained as soon as possible from the individual or a legally authorized surrogate.
27. Both authors and publishers have ethical obligations. In publication of the results of research, the investigators are obliged to preserve the accuracy of the results. Negative as well as positive results should be published or otherwise publicly available. Sources of funding, institutional affiliations and any possible conflicts of interest should be declared in the publication. Reports of experimentation not in accordance with the principles laid down in this Declaration should not be accepted for publication.

C. ADDITIONAL PRINCIPLES FOR MEDICAL RESEARCH COMBINED WITH MEDICAL CARE

28. The physician may combine medical research with medical care, only to the extent that the research is justified by its potential prophylactic, diagnostic or therapeutic value. When medical research is combined with medical care, additional standards apply to protect the patients who are research subjects.
29. The benefits, risks, burdens and effectiveness of a new method should be tested against those of the best current prophylactic, diagnostic, and therapeutic methods. This does not exclude the use of placebo, or no treatment, in studies where no proven prophylactic, diagnostic or therapeutic method exists.1
30. At the conclusion of the study, every patient entered into the study should be assured of access to the best proven prophylactic, diagnostic and therapeutic methods identified by the study.2
31. The physician should fully inform the patient which aspects of the care are related to the research. The refusal of a patient to participate in a study must never interfere with the patient-physician relationship.
32. In the treatment of a patient, where proven prophylactic, diagnostic and therapeutic methods do not exist or have been ineffective, the physician, with informed consent from the patient, must be free to use unproven or new prophylactic, diagnostic and therapeutic measures, if in the physician's judgement it offers hope of saving life, re-establishing health or alleviating suffering. Where possible, these measures should be made the object of research, designed to evaluate their safety and efficacy. In all cases, new information should be recorded and, where appropriate, published. The other relevant guidelines of this Declaration should be followed.

1 **Note of clarification on paragraph 29 of the WMA Declaration of Helsinki**
The WMA hereby reaffirms its position that extreme care must be taken in making use of a placebo-controlled trial and that in general this methodology should only be used in the absence of existing proven therapy. However, a placebo-controlled trial may be ethically acceptable, even if proven therapy is available, under the following circumstances:
- Where for compelling and scientifically sound methodological reasons its use is necessary to determine the efficacy or safety of a prophylactic, diagnostic or therapeutic method; or
- Where a prophylactic, diagnostic or therapeutic method is being investigated for a minor condition and the patients who receive placebo will not be subject to any additional risk of serious or irreversible harm.

All other provisions of the Declaration of Helsinki must be adhered to, especially the need for appropriate ethical and scientific review.

2 **Note of clarification on paragraph 30 of the WMA Declaration of Helsinki**
The WMA hereby reaffirms its position that it is necessary during the study planning process to identify post-trial access by study participants to prophylactic, diagnostic and therapeutic procedures identified as beneficial in the study or access to other appropriate care. Post-trial access arrangements or other care must be described in the study protocol so the ethical review committee may consider such arrangements during its review.

9.10.2004

Appendix IX

WHO Guideline of Confirmation for HIV Tests

TEST-STRATEGIE

WHO, UNAIDS (March 1997)

	Prevalence	Amount of Tests
Diagnosis with clinical Symptoms	**> = 30%**	**1**
	**< 30%**	**2**
Diagnosis without clinical Symptoms	**> 10%**	**2**
	**< = 10%**	**3**
